# An interaction between PRRT2 and Na^+^/K^+^ ATPase contributes to the control of neuronal excitability

**DOI:** 10.1038/s41419-021-03569-z

**Published:** 2021-03-17

**Authors:** Bruno Sterlini, Alessandra Romei, Chiara Parodi, Davide Aprile, Michele Oneto, Anita Aperia, Pierluigi Valente, Flavia Valtorta, Anna Fassio, Pietro Baldelli, Fabio Benfenati, Anna Corradi

**Affiliations:** 1 Department of Experimental Medicine, University of Genova, Viale Benedetto XV, 3, 16132 Genoa, Italy; 2 Center for Synaptic Neuroscience and Technology, Istituto Italiano di Tecnologia, Largo Rosanna Benzi 10, 16132 Genoa, Italy; 3 IRCCS, Ospedale Policlinico San Martino, Largo Rosanna Benzi 10, 16132 Genoa, Italy; 4 Nanoscopy & NIC@IIT, Center for Human Technologies, Istituto Italiano di Tecnologia, via E. Melen 83B, 16152 Genoa, Italy; 5 Science for Life Laboratory, Department of Women and Children’s Health, Karolinska Institute, Stockholm, Sweden; 6 IRCCS San Raffaele Scientific Institute and Vita-Salute University, Via Olgettina 58, 20132 Milan, Italy

**Keywords:** Proteomics, Cellular neuroscience, Molecular neuroscience, Paediatric neurological disorders

## Abstract

Mutations in PRoline Rich Transmembrane protein 2 (PRRT2) cause pleiotropic syndromes including benign infantile epilepsy, paroxysmal kinesigenic dyskinesia, episodic ataxia, that share the paroxysmal character of the clinical manifestations. PRRT2 is a neuronal protein that plays multiple roles in the regulation of neuronal development, excitability, and neurotransmitter release. To better understand the physiopathology of these clinical phenotypes, we investigated PRRT2 interactome in mouse brain by a pulldown-based proteomic approach and identified α1 and α3 Na^+^/K^+^ ATPase (NKA) pumps as major PRRT2-binding proteins. We confirmed PRRT2 and NKA interaction by biochemical approaches and showed their colocalization at neuronal plasma membrane. The acute or constitutive inactivation of PRRT2 had a functional impact on NKA. While PRRT2-deficiency did not modify NKA expression and surface exposure, it caused an increased clustering of α3-NKA on the plasma membrane. Electrophysiological recordings showed that PRRT2-deficiency in primary neurons impaired NKA function during neuronal stimulation without affecting pump activity under resting conditions. Both phenotypes were fully normalized by re-expression of PRRT2 in PRRT2-deficient neurons. In addition, the NKA-dependent afterhyperpolarization that follows high-frequency firing was also reduced in PRRT2-silenced neurons. Taken together, these results demonstrate that PRRT2 is a physiological modulator of NKA function and suggest that an impaired NKA activity contributes to the hyperexcitability phenotype caused by PRRT2 deficiency.

## Introduction

Mutations in the PRRT2 gene cause a variety of paroxysmal disorders including paroxysmal kinesigenic dyskinesia (PKD), benign infantile epilepsy, episodic ataxia, and migraine that can be present alone or in combination. Most patients (>80%) carry the same frameshift mutation (c.649dupC; p.Arg217Profs*8) that generates a precocious stop codon followed by degradation of the mRNA or the protein, leading to haploinsufficiency^1–4^. A few patients bearing PRRT2 mutations in homozygosity or compound heterozygosity display a very severe phenotype that includes most of the isolated disorders, developmental delay and intellectual disability^5^.

PRRT2 is a neuronal type-2 transmembrane protein that is a major determinant of network stability^5–7^. PRRT2 controls synaptic transmission and plasticity and intrinsic excitability^8–10^. PRRT2 knock out (KO) mice are viable and fertile, but show hyperkinetic movements, motor paroxysms, and an increased seizure propensity that are very reminiscent of the human PRRT2-linked pathologies^11^. Particularly relevant is the involvement of the cerebellum, where PRRT2 deficiency in granule cells recapitulates the behavioral phenotype of PRRT2 KO mice^11,12^.

To better understand PRRT2 function and the molecular basis of PRRT2-linked pathologies, we undertook a pulldown-based proteomic approach, aimed at identifying new PRRT2 interacting proteins. The α1 and α3 subunits of Na^+^/K^+^ ATPase pump (NKA) resulted amongst the highest score PRRT2 interactors. The α-NKA subunit is a fundamental polarized and electrogenic pump that transports 3 Na^+^ ions outside the cell and two K^+^ ions inside for each ATP hydrolyzed. Thanks to this activity, NKA is the main responsible for the ion gradient maintenance across the plasma membrane and contributes to the neuronal resting potential^13^.

The NKA α-subunit has the catalytic and ion transport functions and associates with the β-subunit that modulates its membrane exposure and activity. Three isoforms of α-subunit are expressed in brain: while the α1 subunit is ubiquitous, the α3 and α2 subunits are specifically expressed in neurons and glial cells, respectively. Neurons have higher requirements of pumping activity due to resting Na^+^ permeability, firing activity and inward synaptic currents that enrich neurons of Na^+^^14,15^. Previous studies in cell lines showed that the α1-subunit has higher Na^+^ affinity than the α3-subunit. This leads to the hypothesis that, in neurons, the α1 subunit is devoted to basal ion pumping activity, whereas the α3 subunit becomes essential when the intracellular Na^+^ concentration increases during neuronal activity^16–18^.

Mutations in NKA α-isoforms are linked to several pathologies: mutations in the ATP1A2 gene encoding for α2-NKA are linked to familial hemiplegic migraine, whereas mutations in ATP1A3 gene encoding for α3-NKA are associated with a spectrum of paroxysmal neurological phenotypes, such as alternating hemiplegia of childhood (AHC), rapid-onset dystonia Parkinsonism (RDP), capos and epilepsy disorders, that are also related to PRRT2-associated diseases^13,19^. Moreover, recently discovered mutations in the ATP1A1 gene encoding for α1-NKA are linked to refractory seizures and intellectual disability^20^.

In this paper, we show that PRRT2 specifically interacts with both the α1 and the α3 subunits of NKA and that PRRT2 deficiency affects α3-NKA clustering on the plasma membrane and impairs NKA function during neuronal stimulation, a phenotype that was specifically rescued by PRRT2 expression in PRRT2-lacking neurons. These defects in NKA activity may contribute to the hyperexcitability of PRRT2 KO neuronal networks and to the pathogenesis of PRRT2-linked paroxysmal disorders.

## Results

### Proteomic screen with PRRT2 identified α1-NKA and α3-NKA as potential interactors

To perform the proteomic screen for getting insight on the PRRT2 interactome, we generated PRRT2-HA and bacterial alkaline phosphatase (BAP)-HA fusion proteins as specific and nonspecific baits. COS-7 affinity-purified fusion proteins were incubated with total mouse brain extracts, pulled down along with their putative interacting proteins and resolved by SDS–PAGE (Supplementary Fig. 1A, B). We focused on a region of the Coomassie-stained gels spanning between 95 and 130 kDa, where we discriminated few distinct bands in the PRRT2-HA lane that were virtually absent in the BAP-HA lane (Supplementary Fig. 1A, insets). We excised the corresponding bands from both the PRRT2-HA and BAP-HA lanes and subjected them to mass spectrometry.

The proteins specifically identified in the PRRT2-HA sample with the highest number of hits are shown in Table 1 (for an overall list of putative interactors see Supplementary Table 1). Gene Ontology (GO) analysis by Panther^21^ revealed that the most represented protein classes in the PRRT2 interactome were transporters (α-NKAs, sarcoplasmic/endoplasmic reticulum Ca^2+^ ATPase 2, plasma membrane Ca^2+^-transporting ATPase 1), nuclear transporters (Exportin-1 and Importin-7) and membrane-trafficking proteins (AP-2 complex subunit α1, clathrin heavy chain 1, dynamin 1, microtubule-associated protein 6) (Supplementary Fig. 1C). Among the best candidate interactors, α1, α2, and α3-NKA subunits displayed a high number of peptides that covered all the protein length (Supplementary Fig. 1D). The three subunits have 87% sequence identity, but show differential expression profiles, ubiquitous for α1, glial-specific for α2, and neuron-specific for α3. In our proteomic screen, we used total mouse brain extracts including neuronal and glial proteins. However, since PRRT2 is a neuron-specific protein lacking extracellular domains, the binding to glial proteins is unlikely. Therefore, we considered only neuronal proteins as putative candidate interactors and excluded α2-NKA and glial proteins from further analysis. Given the crucial role played by α3 and α1-NKA in neuronal function and considering that their mutations are associated with neuronal hyperexcitability and PRRT2-like neurological phenotypes, we decided to better characterize these putative interactions.

**Table 1:** List of best interacting proteins obtained by mass spectrometry analysis.

List of best interacting proteins	Accession number	MW (kDa)	Hits
Cullin-associated NEDD8-dissociated protein 1	Q6ZQ38	136	19
Sodium/potassium-transporting ATPase subunit alpha-2	Q6PIE5	112	18
Exportin-2	Q9ERK4	110	16
Sarcoplasmic/endoplasmic reticulum calcium ATPase 2	O55143	115	16
Puromycin-sensitive aminopeptidase	Q11011	103	15
Sodium/potassium-transporting ATPase subunit alpha-1	Q8VDN2	113	11
Sodium/potassium-transporting ATPase subunit alpha-3	Q6PIC6	112	10
Exportin-1	Q6P5F9	123	10
Importin-7	Q9EPL8	119	9
Plasma membrane calcium-transporting ATPase 1	G5E829	135	8
Exportin-7	Q9EPK7	124	8
Importin-9	Q91YE6	116	7
Putative uncharacterized protein	Q3V1M8	142	7
AP-2 complex subunit alpha-1	P17426	108	6
Microtubule-associated protein 6	Q7TSJ2	96	6
Importin-5	Q8BKC5	124	6
Splicing factor 3B subunit 1	Q99NB9	146	6
Drebrin	Q9QXS6	77	5
DNA damage-binding protein 1	Q3U1J4	127	5

The numbers in the “Hits” column refer to the mean amounts of unique peptides identified in three independent experiments. α3-NKA and α1-NKA were among the major interactors. See Supplementary Table 1 for a complete list of interactors.

### PRRT2 associates with α3-NKA and α1-NKA in brain extracts

**Fig. 1:**
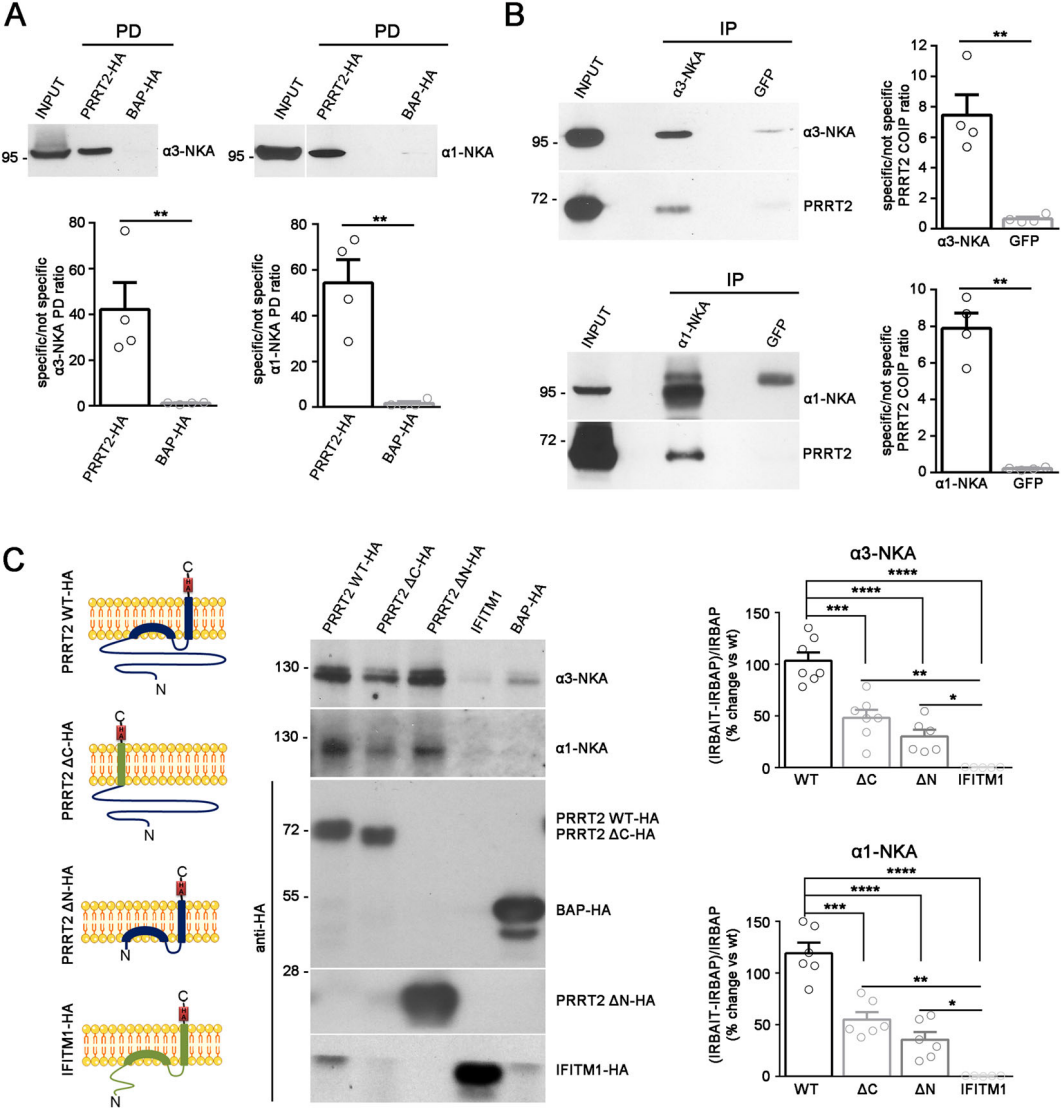
PRRT2 interacts with α3-NKA and α1-NKA subunits. **A** *Pulldown of α3-NKA and α1-NKA by PRRT2*: Affinity-purified PRRT2-HA or BAP-HA were incubated with total mouse brain extracts. After pulldown (PD), pellets were solubilized and subjected to western blotting with α3-NKA and α1-NKA antibodies. Mouse brain lysates incubated with PRRT2-HA showed specific immunoreactivity for α3-NKA and α1-NKA in the precipitates, which were not detected with BAP-HA. *Top:* Representative immunoblots are shown. *Bottom:* Quantification of the α3-NKA and α1-NKA immunoreactivities in the pulled down samples normalized to the BAP-HA control (means ± SEM, *n* = 4 independent experiments, **p < 0.01; unpaired Student’s *t*-test). Input, 10 μg total extract. **B** *Co-immunoprecipitation of PRRT2 with α3-NKA and α1-NKA antibodies*: Detergent extracts of mouse brain were immunoprecipitated (IP) with monoclonal antibodies (mAbs) to α3-NKA, α1-NKA or GFP (used as a control), as indicated. After the electrophoretic separation of the immunocomplexes and western blotting, membranes were probed with α3-NKA or α1-NKA antibodies to test the immunoprecipitation efficiency, as well as with polyclonal PRRT2 antibodies to test for co-immunoprecipitation. *Left:* Representative immunoblots are shown. *Right:* Quantification of the PRRT2 immunoreactive signal in the immunoprecipitated samples normalized to the GFP nonspecific signal (means ± SEM, *n* = 4 independent experiments, **p < 0.01; unpaired Student’s *t*-test). Input, 10 μg *t*otal extract. **C** *Both the cytosolic and membrane-associated domains of PRRT2 are necessary for the interaction with NKA. Left:* Schematics of the PRRT2 domain constructs. PRRT2ΔC-HA is a chimeric protein composed of the cytoplasmic domain of PRRT2 (violet) anchored to the membrane by the transmembrane domain of IFITM1 (green). PRRT2ΔN-HA is composed of the transmembrane domain of PRRT2. The IFITM1 protein (green) was used as a control. *Middle:* COS-7 cells were co-transfected with full length PRRT2 WT-HA, PRRT2ΔC-HA, PRRT2ΔN-HA, IFITM1-HA or BAP-HA constructs and either α3-NKA-SEP or α1-NKA-SEP constructs. Samples were immunoprecipitated by anti-HA beads (IP) and analyzed by western blotting with antibodies to α3-NKA, α1-NKA and HA. Representative immunoblots are shown. Horizontal white lines in the blot indicate that proteins of different molecular mass were separated on the same original gel and subjected to immunoblotting assays in distinct nitrocellulose strips. *Right:* Densitometric analysis of the fluorograms obtained in the linear range of the emulsion response. The immunoreactivity (IR) of the bait-specific pulldown with respect to the non-specific BAP control [(IRBAIT–IRBAP)/IRBAP] was calculated, normalized by the amount of each BAIT, and expressed in percent of the PRRT2 WT-HA. Means ± SEM of *n* = 6 independent experiments; *p < 0.05, **p < 0.01, ***p < 0.001, ****p < 0.0001; one-way ANOVA/Tukey’s tests.

To confirm the specificity of the interactions, we performed pulldown experiments in which we analyzed the presence of α3/α1-NKA in PRRT2 immuno-isolated fractions from total brain extracts by western blotting using specific α3-NKA and α1-NKA antibodies. As shown in Fig. 1A, a significant pulldown of both α3 and α1-NKA was observed. Moreover, immunoprecipitations from total brain extracts of the endogenous α3-NKA and α1-NKA isoforms with specific antibodies efficiently co-immunoprecipitated PRRT2 (Fig. 1B). These data show that PRRT2 associates with both α3 and α1-NKA, confirming mass spectrometry results.

PRRT2 is a type-2 membrane protein with two membrane α helices, one of which spans the plasma membrane, and a cytosolic less structured domain including a proline-rich region^6^. Thus, to restrict the NKA interaction sites on PRRT2, we performed a pulldown experiment using either the PRRT2 membrane-associated domain (ΔN)^6^ or the PRRT2 cytosolic domain (ΔC) as a bait, as schematized in Fig. 1C. To anchor the PRRT2 cytosolic domain to the plasma membrane, we used the membrane-associated domain of interferon-induced transmembrane protein 1 (IFITM1), a membrane protein that has a topology similar to that of PRRT2 and used IFITM1 as negative control. Both PRRT2 domains were able to significantly pulldown both α3-NKA and α1-NKA with respect to the control. However, their binding was significantly lower as compared to full length PRRT2, suggesting that both membrane-associated and cytosolic domains contribute to the efficient binding of PRRT2 to NKAs (Fig. 1C).

### PRRT2 and NKA colocalize on the neuronal plasma membrane

**Fig. 2:**
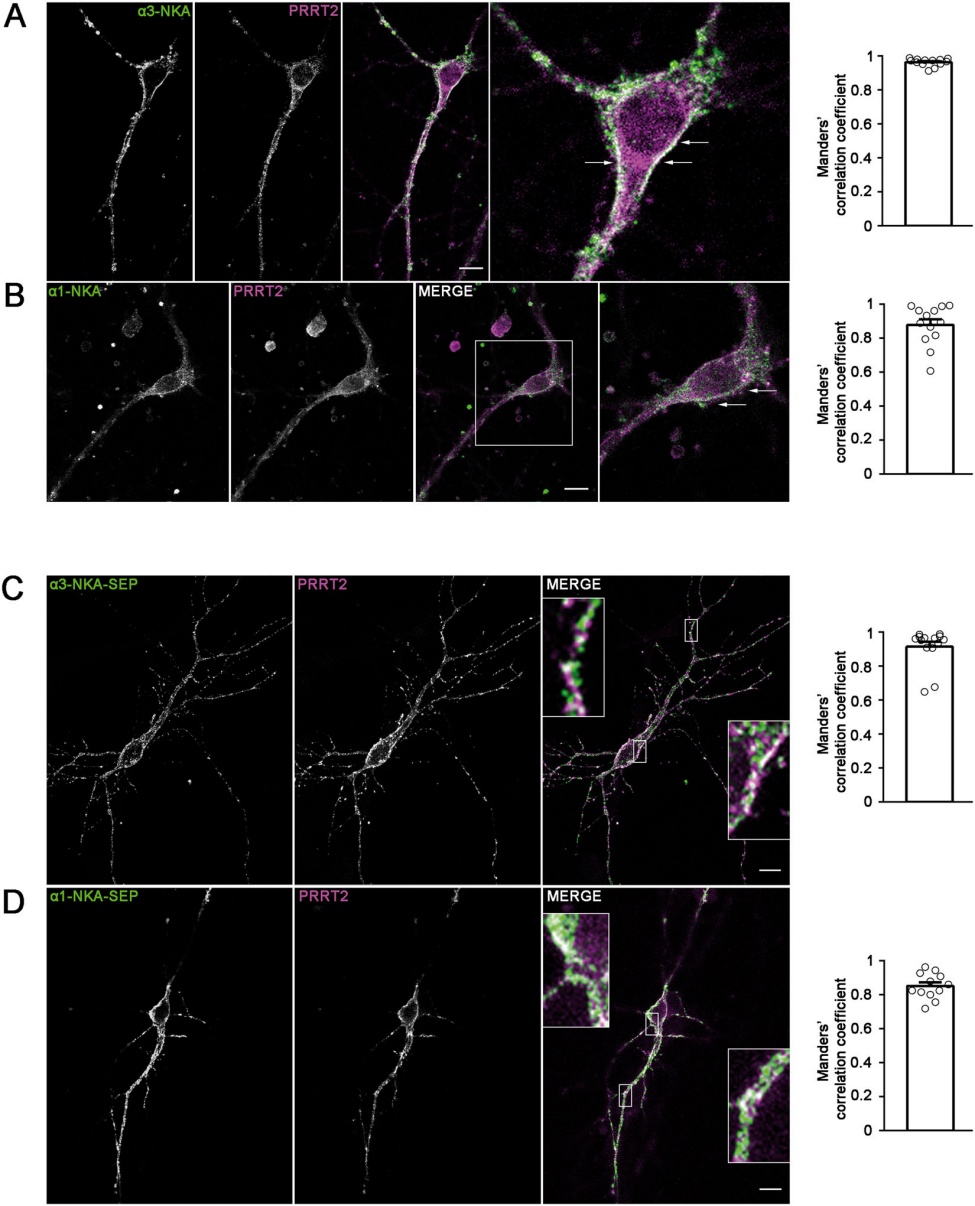
Co-localization of α3-NKA and α1-NKA with PRRT2 in primary hippocampal neurons. **A**, **B** Hippocampal neurons (DIV 14) were stained for endogenous PRRT2 and either α3-NKA (**A**) or α1-NKA (**B**). *Left:* Representative confocal images show the overlapping staining of NKA isoforms and PRRT2 (magnified in the insets). Scale bar, 10 μm. *Right:* Quantitative evaluation of the extent of the codistribution between PRRT2 and NKA isoforms using the Manders’ correlation coefficient expressing the PRRT2/NAK overlap area in percent of the NAK immunoreactive area (0.96 ± 0.06 and 0.87 ± 0.04 for α3-NKA/PRRT2 and α1-NKA/PRRT2, respectively; *n* = 15 images from three independent experiments). **C**, **D** Hippocampal neurons transfected with PRRT2-HA and either α3-NKA-SEP (**C**) or α1-NKA-SEP (**D**) were live-labeled (DIV 14) with anti-HA and anti-GFP antibodies to stain the surface-exposed domains of the proteins. *Left*: The images show the largely overlapping staining of PRRT2 with both α3-NKA-SEP and α1-NKA-SEP (magnified in the insets). Scale bar, 10 μm. *Right:* Quantitative evaluation of the extent of the codistribution between PRRT2-HA and the NKA-SEP isoforms using the Manders’ correlation coefficient expressing the PRRT2/NAK overlap area in percent of the area of NAK fluorescence (0.91 ± 0.04 and 0.84 ± 0.03 for α3-NKA/PRRT2 and α1-NKA/PRRT2, respectively; *n* = 15 images from three independent experiments).

To ascertain whether the PRRT2–NKA interaction was supported by the co-localization of the two proteins in neurons, we performed double immunofluorescence with PRRT2 and α3/α1-NKA antibodies in primary hippocampal neuronal cultures. The α3-NKA subunit was confined to the plasma membrane of the neuronal soma and processes, while PRRT2 was present both on the membrane and inside the cell (Fig. 2A). A clear co-localization between α3-NKA and PRRT2 at the plasma membrane was present, as demonstrated by the high Manders’ correlation coefficient describing the ratio between the overlap area and the total area of α3-NKA immunoreactivity (Fig. 2A, right). A similar distribution was found for endogenous α1-NKA and for its co-localization with PRRT2 (Fig. 2B).

To confirm the co-localization of PRRT2 and either α1-NKA or α3-NKA, we co-expressed PRRT2-HA and either α3-NKA or α1-NKA fused to superecliptic-pHluorin (SEP)^22^ in primary hippocampal neurons at 10 days in vitro (DIV) and analyzed their surface localization on the plasma membrane by live labeling with anti-HA/GFP antibodies at 14 DIV. Both α-subunits of NKA were sharply expressed on the plasma membrane and significantly colocalized with PRRT2 in co-transfection assays (Fig. 2C, D). The quantitative evaluation of the colocalization between PRRT2 and α3/α1-NKA subunits using the Manders’ correlation coefficient confirmed that the PRRT2/NKA overlap accounts for the vast majority of the NKA immunoreactive area for both NKA isoforms (Fig. 2C, D).

### PRRT2 does not affect NKA expression level at the membrane surface

To investigate the functional significance of the α1/α3-NKA interaction with PRRT2, we first checked whether the lack of PRRT2 affects the expression of NKA by comparing protein levels in various brain regions from WT and PRRT2 KO mice. However, we could not find any alteration of either α3-NKA or α1-NKA overall protein expression in the brain regions analyzed (Supplementary Fig. 2A), as well as any quantitative differences in the specific immunoreactivity of both α3-NKA and α1-NKA in PRRT2 KO primary hippocampal neurons as compared to WT neurons (Supplementary Fig. 2B).

**Fig. 3:**
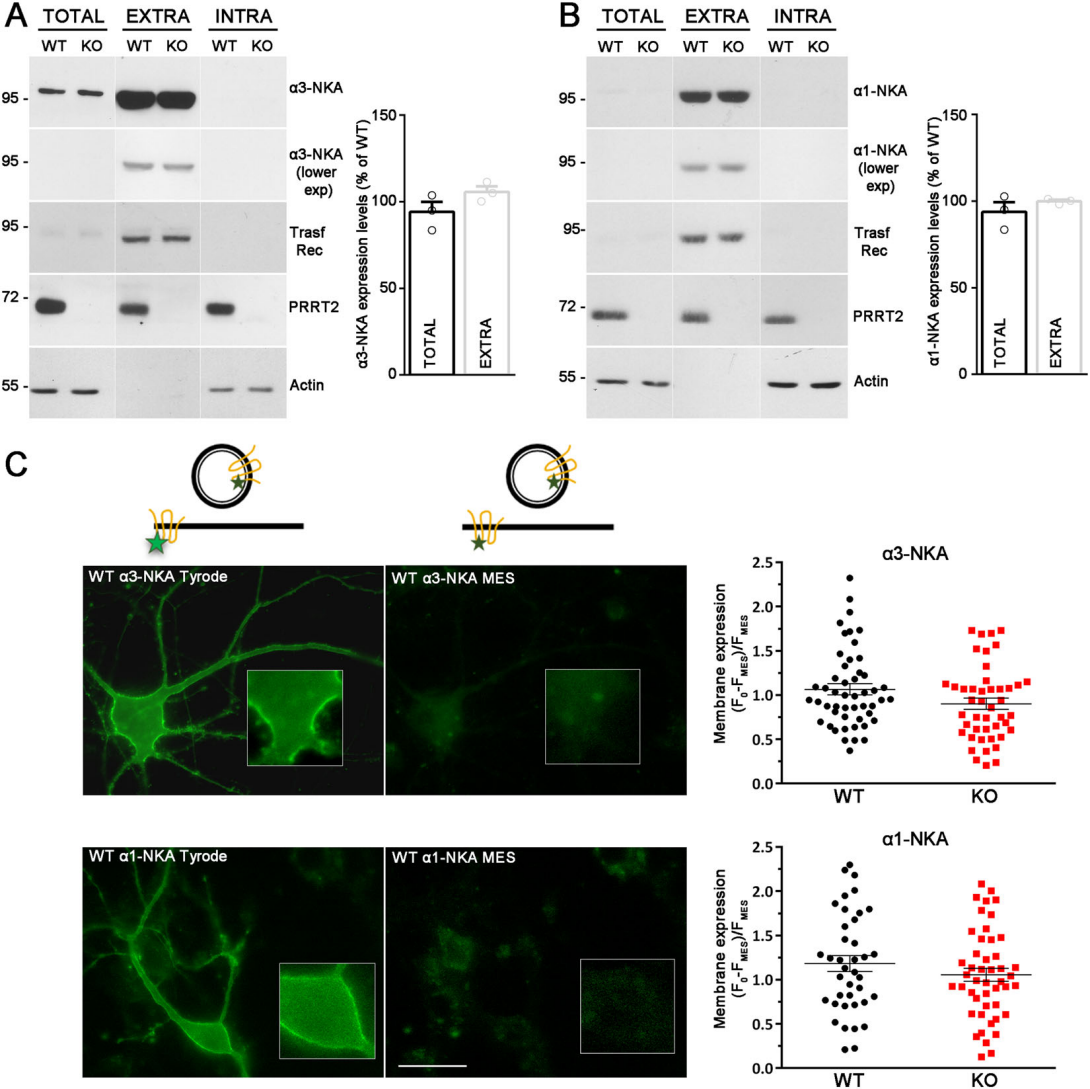
PRRT2 does not affect NKA expression level at the membrane surface. **A**, **B** *Left:* Representative immunoblots of cell surface biotinylation performed in WT and PRRT2 KO hippocampal neurons. Total lysates (TOTAL), biotinylated (cell surface, EXTRA), and non-biotinylated (intracellular, INTRA) fractions were analyzed by western blotting with antibodies to PRRT2 and either α3-NKA (**A**) or α1-NKA (**B**). Antibodies to transferrin receptor (Transf Rec) and actin were used as markers of cell surface and intracellular fractions, respectively. Vertical lines in the blot indicate that the lanes were on the same gel but have been repositioned in the figure. *Right:* Total and cell surface α3-NKA (**A**) and α1-NKA (**B**) immunoreactivities are expressed in percent of the respective WT value after normalization to Transferrin receptor (for the EXTRA fraction) or actin (for the INTRA fraction). Means ± SEM of *n* = 3 independent experiments; unpaired Mann–Whitney’s *U*-test. **C**, **D** *Left*: Representative images of a WT neuron transfected with α3-NKA-SEP (**C**) or α1-NKA-SEP (**D**) perfused with Tyrode followed by MES buffers (scale bar, 20 μm). Insets show higher magnification of the fluorescence at the plasma membrane where ROIs were measured. The cartoons shown on top depict the SEP state under Tyrode and MES buffers, respectively. The green stars represent fluorescent emission of the chimeric protein, which is quenched at acidic pH, i.e., in acidic intracellular compartments and in the presence of extracellular MES buffer. *Right:* Membrane expression of α3-NKA-SEP (**C**) and α1-NKA-SEP (**D**) in WT and PRRT2 KO neurons, calculated as the normalized difference between fluorescence in Tyrode (*F*_0_) and fluorescence in MES (*F*_MES_) as [(*F*_0_−*F*_MES_)/*F*_MES_]. Data are means ± SEM of 40–51 neurons, obtained from *n* = 3 independent preparations for WT and PRRT2 KO, respectively.

As we have shown that PRRT2 modulates the surface expression of Na_V_ channels^9^, we addressed the possibility that PRRT2 may influence the amount of α1/α3-NKA expressed at the plasma membrane. We therefore evaluated the membrane expression of α1-NKA and α3-NKA by surface protein biotinylation. However, no significant differences in the membrane-exposed NKA isoforms were observed between WT and PRRT2 KO neurons (Fig. 3A, B).

We next examined the NKA trafficking to the membrane surface in live cells by expressing the pH-sensitive NKA-SEP constructs^22^. Hippocampal WT and PRRT2 KO neurons (14 DIV) transfected with either α3-NKA-SEP or α1-NKA-SEP were perfused with standard extracellular solution (Tyrode) followed by acidic extracellular buffer (MES) to quench the membrane-exposed fluorescence. Then, we calculated the fraction of extracellularly exposed NKA as the normalized difference between the fluorescence signals in Tyrode and MES (*F*_0_ and *F*_MES_, respectively; Supplementary Fig. 3) measured in linear regions of interests (ROIs) of the plasma membrane. Consistent with the results obtained by surface biotinylation, no differences were observed in the membrane expression of either α3-NKA-SEP or α1-NKA-SEP in the absence of PRRT2 (Fig. 3C, D). Taken together, the results indicate that PRRT2 does not significantly affect the exposure of α3-NKA and α1-NKA at the plasma membrane and their trafficking between the intracellular compartments and the plasma membrane

### PRRT2 influences α3-NKA clustering at the membrane surface

**Fig. 4:**
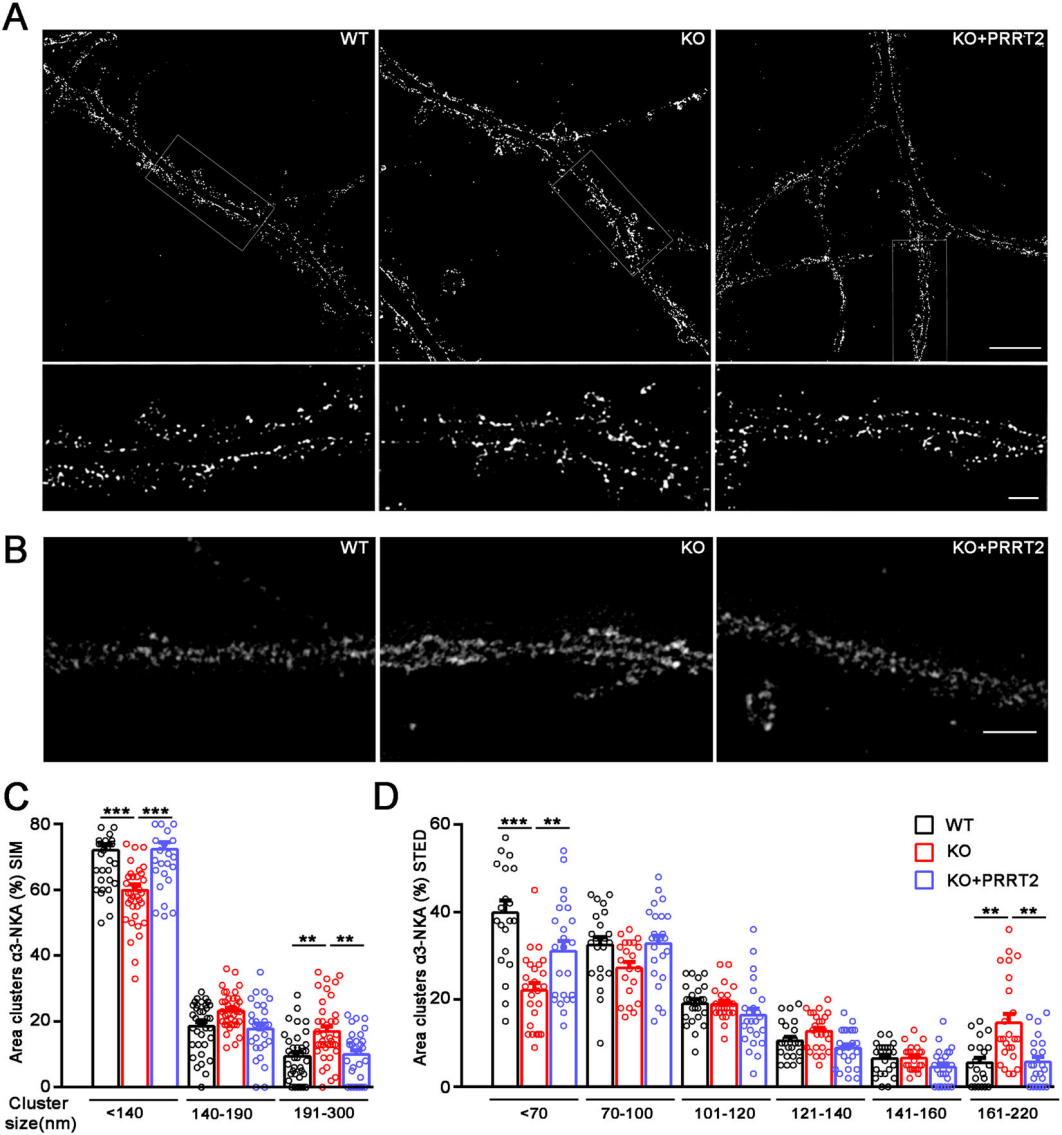
PRRT2 affects α3-NKA clustering at the membrane surface. **A**, **B** Representative 3D-SIM (**A**) and STED (**B**) images of hippocampal neurons (DIV 14-17) stained for α3-NKA. The images highlight an altered clustering of α3-NKA along the plasma membrane in PRRT2 KO neurons as compared to WT neurons that is rescued by expression of PRRT2 in PRRT2 KO neurons (KO + PRRT2). Scale bar, 5 μm; insets: 1 μm. **C**, **D** Size distribution of α3-NKA membrane clusters. Clusters were subdivided into bins on the basis of their size as indicated. The histograms (means ± SEM) show the area occupied by each bin of α3-NKA clusters expressed in percent of the total immunoreactive area. Both 3D-SIM (**C**) and STED (**D**) microscopy showed a significant increase in the area of largest clusters and a reciprocal decrease in the area of smallest clusters along the soma and the dendritic membrane in PRRT2 KO neurons (red bars) with respect to WT neurons (black bars). The change was fully rescued by re-expression of PRRT2 in PRRT2 KO neurons (KO + PRRT2, blue bars). Data refer to *n* = 35 (3D-SIM) and 25 (STED) neurons per genotype, from *n* = 3 independent preparations (3 coverslips/preparation/genotype). **p < 0.01, ***p < 0.001; one-way ANOVA/Bonferroni tests.

Super-resolution microscopy has revealed that α3-NKA is organized in nanoclusters at the plasma membrane^22,23^. In the absence of an effect on membrane exposure, we addressed the possibility that PRRT2 is involved in the regulation of α3-NKA clustering. To this aim, we used high-resolution structured illumination microscopy (3D-SIM). 3D-SIM is able to resolve objects beyond the diffraction limit by illuminating with multiple interfering beams of light (resolution limit: 120 nm). When the α3-NKA immunostaining was analyzed in WT and PRRT2 KO neurons at this spatial resolution, α3-NKA nanoclusters were clearly detectable, allowing a quantification of their distribution along MAP2-stained neuronal cell bodies and dendrites (Fig. 4A and Supplementary Fig. 4A). Both the total number and the total area of α3-NKA nanoclusters were not changed in PRRT2 KO neurons, confirming that the overall surface expression was not affected by PRRT2 deficiency (Supplementary Fig. 4A, B). However, when α3-NKA clusters were grouped into three bins of different size (diameter: <140, 141–190, and 190–300 nm), the area of the larger clusters was significantly higher in PRRT2 KO compared to WT neurons and, conversely, the area and number of smaller clusters were significantly decreased in PRRT2 KO neurons (Fig. 4C, Supplementary Fig. 4C). To ensure that the observed phenotypic changes were specifically dependent on the lack of PRRT2, we rescued its expression in PRRT2 KO neurons by transduction with lentiviruses encoding mouse PRRT2. Strikingly, re-expression of PRRT2 normalized nanocluster number and size to the WT levels, suggesting a specific effect of PRRT2 on α3-NKA clustering (Fig. 4A, C). To analyze α3-NKA clustering in greater detail, we used stimulated emission depletion (STED) nanoscopy, a distinct super-resolution approach. The analysis of STED images confirmed the alteration of α3-NKA clustering observed with 3D-SIM in PRRT2 KO neurons, with an increased area of larger clusters and a correspondingly decreased area of the smaller ones (Fig. 4B, D). The enhanced resolution of STED microscopy allowed us to analyze the smaller clusters (< 120 nm diameter) that were not resolved by 3D-SIM. Interestingly, only nanoclusters smaller than 70 nm were significantly decreased in PRRT2 KO neurons, suggesting that the smallest nanoclusters are the ones that aggregate to form larger clusters and shift the α3-NKA membrane distribution to larger nanoclusters.

### PRRT2-deficient primary hippocampal neurons show an activity-dependent reduction of NKA activity

The new reported interaction of PRRT2 with α3-NKA and α1-NKA and the abnormal α3-NKA clustering that was recently associated with a decreased efficiency of Na^+^ extrusion, might underlie an altered pump function in neurons that lack PRRT2 expression^15,23–25^. Therefore, we evaluated both the resting and the activity-dependent NKA activity by whole-cell patch-clamp recordings in primary excitatory neurons in which PRRT2 had been constitutively deleted (PRRT2 KO) or acutely silenced by RNA interference (PRRT2 KD).

The activity of NKA at rest was studied by maintaining neurons at a holding potential of −70 mV and inhibiting the pump with ouabain (1 mM), a concentration able to block both α3 and α1 NKA isoforms^17^. Bath application of ouabain elicited a tonic inward current that was normalized by cell capacitance and expressed as current density (*J*, p*A*/p*F*). The density of the ouabain-sensitive current was not affected by PRRT2 deletion, either acute or chronic (Supplementary Fig. 5A, B). Consistently, the steady-state membrane depolarization induced by NKA blockade remained unchanged, irrespective of either chronic or acute PRRT2 deficiency (Supplementary Fig. 5C, D).

**Fig. 5:**
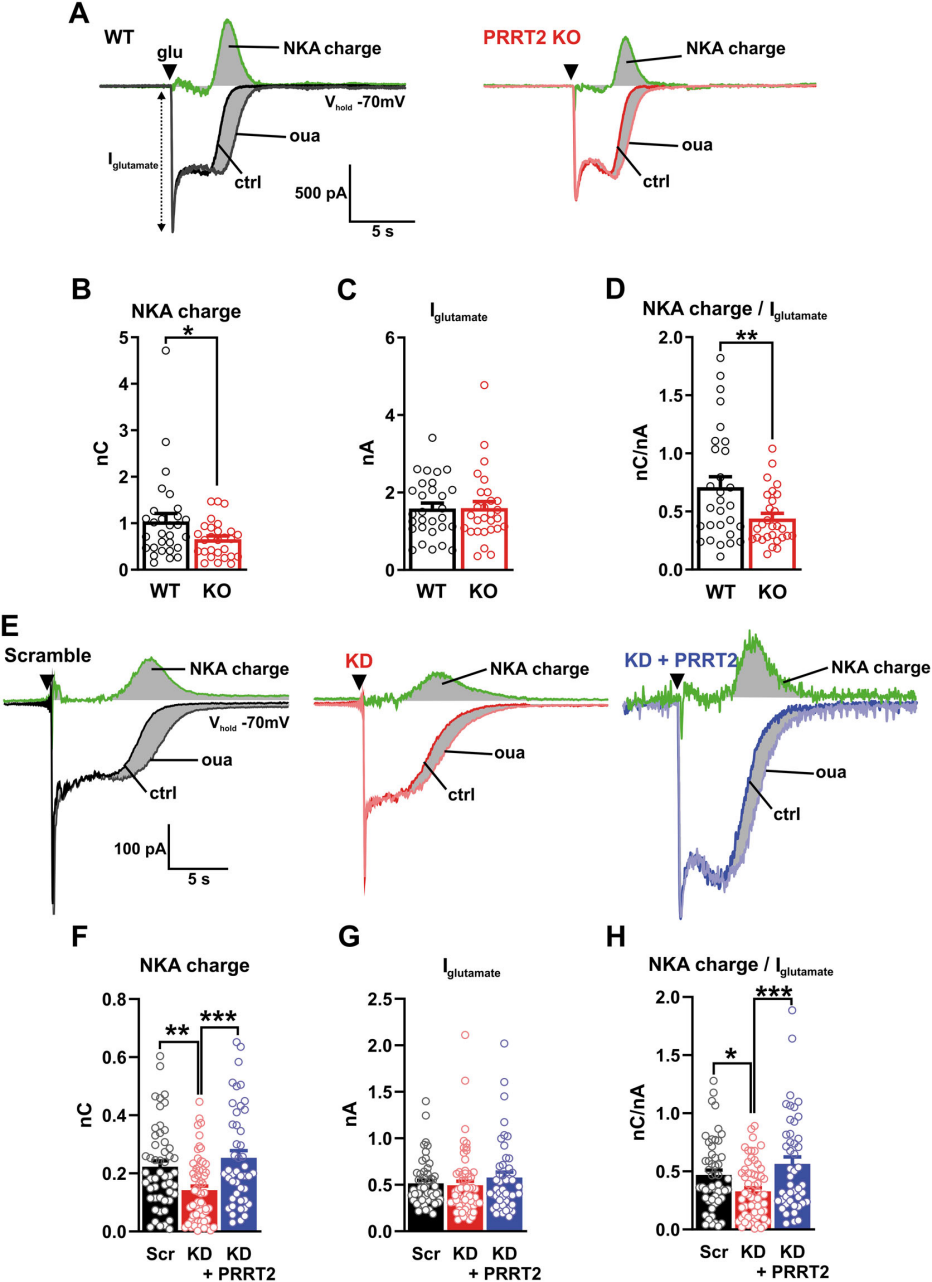
Glutamate-evoked NKA activity is reduced in PRRT2-deficient neurons. **A** Representative traces from WT (black; *left*) and PRRT2 KO (red; *right)* primary hippocampal neurons show the response to glutamate puffs performed under control conditions (ctrl) or under blockade of the NKA with 1 mM ouabain (oua). Black triangles show the time of the application of the glutamate puff (glu). Digital subtraction of the response obtained in the presence of ouabain from the control response yields the NKA activity (green traces). The integrated area under this ouabain-sensitive current (gray-shaded area) defines the NKA charge. **B**–**D** NKA charge (**B**), glutamate puff-induced current (**C**), and NKA charge normalized by the amplitude of the glutamate current (**D**) for WT and PRRT2 KO primary neurons (*n* = 29 and 28 neurons for WT and PRRT2 KO, respectively, from three independent neuronal preparations). **E** Primary hippocampal neurons were subjected to acute PRRT2 knockdown (KD) by RNA interference and to rescue of PRRT2 expression by transduction with a Sh-resistant PRRT2 construct. Representative traces recorded from neurons 7 days post-infection with Scramble/Control Cherry (Scr; black; *left*) or Sh4/Control Cherry (KD; red; *middle*) or Sh4/PRRT2-Cherry (KD + PRRT2; blue; *right*) upon glutamate puffs performed in the absence (ctrl) or presence of 1 mM ouabain (oua). Black triangles mark the time of glutamate application. Green traces indicate the NKA activity. **F**–**H** NKA charge (**F**), glutamate puff-induced current (**G**) and NKA charge normalized by the amplitude of the glutamate current (**H**) for Scr, PRRT2 KD, and KD + PRRT2 neurons (*n* = 52, 61, and 46 neurons for Scr, PRRT2 KD and KD + PRRT2, respectively, from six independent neuronal preparations). Bar plots show the means ± SEM with superimposed individual experimental points. *p < 0.05, **p < 0.01, ***p < 0.001; one-way ANOVA/Bonferroni’s tests.

Under resting conditions, a fraction of membrane-bound NKA is inactive and therefore insensitive to pharmacological inhibition^26,27^. With the aim of boosting NKAs activity, the intracellular Na^+^ concentration was increased by focal application of glutamate (250 µM for 250 ms). Such stimulation was demonstrated to induce pump activation in hippocampal cells^28–30^. Glutamate application elicited a fast peak of inward current that, after a rapid decay to a steady-state level, recovered the baseline value in all recorded neurons. We estimated NKA activity by measuring the charge of the ouabain-sensitive current, obtained by digitally subtracting the ouabain-insensitive current from the total evoked current (Fig. 5A, E). To determine NKA activity independently of any variability in the Na^+^ influx induced by glutamate application, the NKA charge was normalized by the peak amplitude of glutamate current (*I*_glutamate_). Both the NKA charge and the normalized NKA charge analysis revealed that both genetic deletion of the *Prrt*2 gene (Fig. 5A–D) and the acute silencing of PRRT2 expression (Fig. 5E–H) induced a significant reduction of NKA activity. Interestingly, the expression of Sh-resistant PRRT2 completely rescued the impairment in NKA activity of silenced neurons, indicating that PRRT2 acts as a modulator of NKA activity under conditions of neuronal stimulation (Fig. 5E–H).

### Acute PRRT2 deficiency impacts on the afterhyperpolarization (AHP) following high-frequency trains

In many neuronal populations, NKA provides an intrinsic negative feedback to inhibit excitability after prolonged firing activity, as a result of the intracellular Na^+^ buildup during the trains^31–33^. Accordingly, high-frequency trains of ≥5 spikes are known to generate a long-lasting AHP in neocortical and CA1 hippocampal neurons^33,34^. While an early and small component of this AHP is generated by Ca^2+^-gated K^+^ channels, the predominant K^+^(Ca^2+^)-independent AHP component is operated by the NKA (NKA-AHP)^33,34^.

**Fig. 6:**
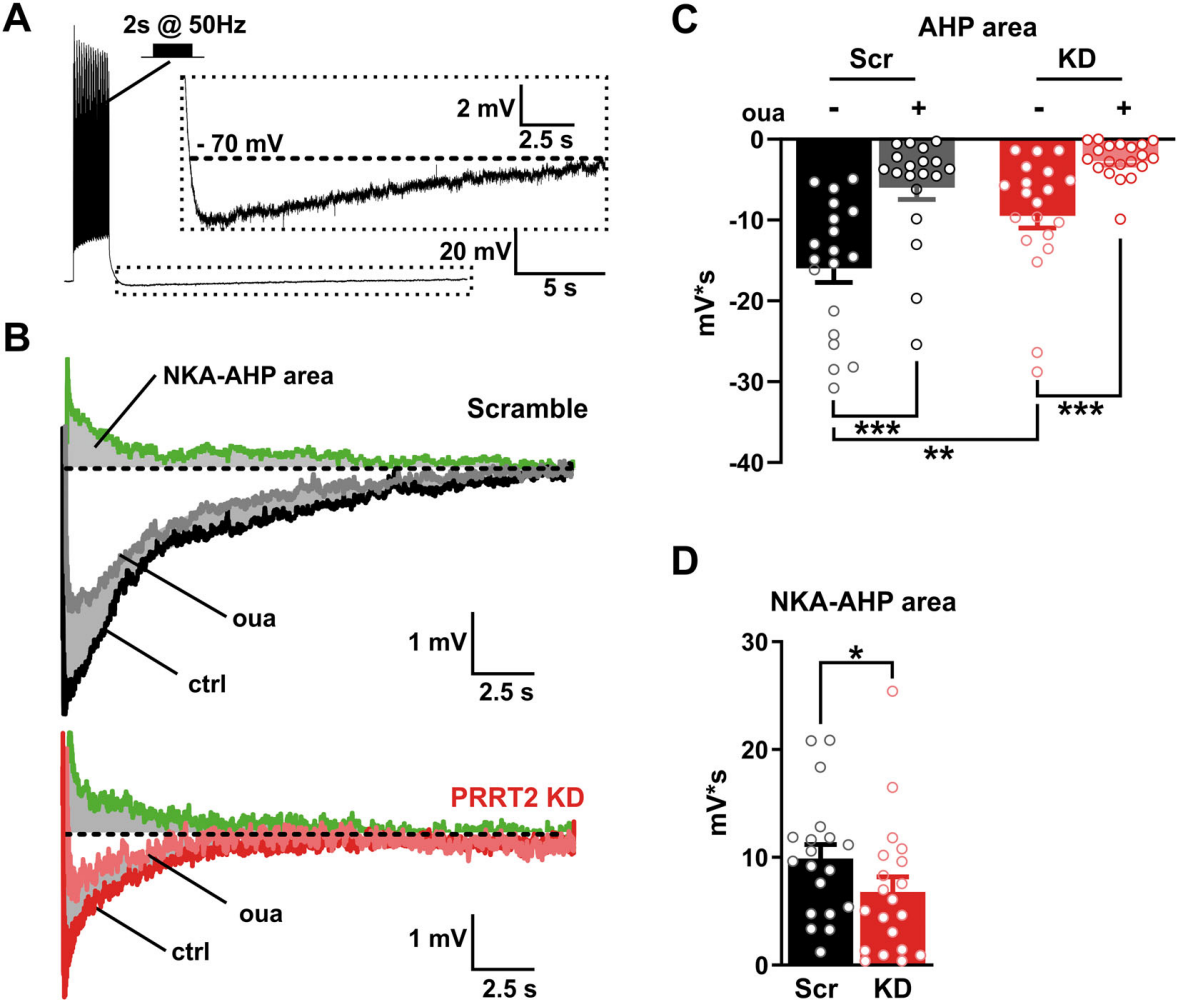
Acute PRRT2 deletion decreases the activation of NKA evoked by high-frequency stimulation. **A** AHP induction protocol. Neurons were kept in whole-cell current-clamp configuration and APs were evoked by delivering supra-threshold current pulses at 50 Hz for 2 s. Inset: expanded view of the AHP phase indicated by the dashed box. The black dashed lines indicate the holding potential (−70 mV). **B** Representative AHP traces recorded from Scr (top) and PRRT2 KD (bottom) neurons before (ctrl) and after the application of ouabain (oua, 1 mM). The NKA-induced AHP was obtained by digital subtraction of the response in the presence of ouabain from the control response (green traces). Gray-shaded areas indicate the NKA-induced AHP component. **C** The areas under the AHP waveform, baselined to −70 mV and within a time window of 20 s after the train, were measured in Scr and PRRT2 KD neurons before and after ouabain blockade. **D** The areas of the NKA-dependent AHP in Scr and PRRT2 KD neurons were obtained from the difference between individual AHP areas before and after ouabain blockade (*n* = 19 and 20 for Scr and PRRT2 KD, respectively, from three independent neuronal preparations). Bar plots show the means ± SEM with superimposed individual experimental points. *p < 0.05, **p < 0.01, ***p < 0.001; two-way ANOVA/Šídák (**C**); unpaired Student’s *t*-test (**D**).

To obtain a further and independent readout of NKA activity in PRRT2-depleted neurons, AHPs were elicited stimulating neurons with a train of action potentials (APs; 50 Hz for 2 s)^34^ (Fig. 6A). The acute silencing of PRRT2 markedly reduced the AHP magnitude under control conditions (Fig. 6B). The specific contribution of NKA to AHP was estimated by measuring the area of the ouabain-sensitive AHP, obtained as the difference between AHP areas recorded under control conditions and those under ouabain treatment. This analysis confirmed that the ouabain-sensitive pump activity during AHP was significantly decreased by the acute deletion of PRRT2 (Fig. 6C, D). These data, obtained with a distinct functional protocol, strongly support the existence of a functional modulation of PRRT2 on NKA activity evoked by neuronal stimulation.

## Discussion

Gaining insights into PRRT2 function is of primary importance to understand the pathogenesis and improve the treatment of the pleiotropic paroxysmal disorders associated with PRRT2 mutations. To investigate PRRT2 function, we identified several putative interacting proteins by a pulldown-based proteomic approach. Among them, α3 and α1-NKA pumps displayed high scores and were particularly interesting hits, given their crucial physiological role and the association of NKA mutations with a spectrum of paroxysmal phenotypes largely overlapping with PRRT2-linked diseases^13,19^. The presence of an NKA/PRRT2 interaction was confirmed biochemically and their cell and membrane distribution were found to overlap considerably, supporting the idea of a physiologically meaningful interaction.

We and others previously demonstrated that PRRT2 interacts with several protein complexes in the neuron. PRRT2 modulates intrinsic neuronal excitability by interacting with α-subunit of Na_V_1.2/1.6 channels^9^. In addition, it plays a role in neurotransmitter release and synaptic plasticity by interacting with the SNARE complex and the Ca^2+^ sensors synaptotagmin1/2 proteins^2,8,35^, and regulates synapse and spine formation by modulating actin dynamics^36^. Moreover, more than one proteomic study identified PRRT2 as a potential AMPA receptor auxiliary protein^37–40^. Taken together all these experimental results suggest that PRRT2 is a component of important macromolecular complexes both in the axon and at the synaptic level, potentially regulating the trafficking of integral membrane proteins between the plasma membrane and the intracellular compartment.

PRRT2 has been defined as “*chameleon protein*”^41^, because it is able to interact with a wide range of proteins. This ability is probably due to its intracellular intrinsically disordered region, as evaluated by two distinct disorder predictors (IUPred and ANCHOR)^42^. Interestingly, several intrinsically disordered proteins function in signaling networks as hubs that integrate multiple cues and link diverse signaling pathways. Their interactions tend to be of low affinity, and thereby can easily be turned on and off, yet they can be highly specific, a feature that is often coupled to regulatory functions^43–45^. As in the case of other adapter proteins involved in signal transduction, PRRT2 might act by assembling arrays of actuators through interactions with its proline-rich intracellular domain. Indeed, we have shown that this PRRT2 domain is implicated in the interaction with α3-NKA and α1-NKA. Recent work has shown the capability of scaffold proteins (such as synapsins, SYD-2, ELKS-1) to trigger liquid–liquid phase separation (LLPS), thus participating in the assembly of integral membrane protein clusters and supramolecular complexes, such as the active zone or the synaptic vesicle clusters^46–49^. It is tempting to speculate that also PRRT2, thanks to its cytoplasmic disordered region, can initiate LLPS in neurons and this way regulate the trafficking and clustering of multiple complex membrane proteins including ion channel and pumps, such as NKA.

The functional counterpart of the NKA/PRRT2 interaction is distinct from the previously reported effect on Na_V_ channel membrane exposure. Indeed, no effects of PRRT2 deficiency were observed on NKA expression, membrane exposure or trafficking between membrane and cytosol. It has been shown that the neuron-specific isoform α3-NKA is not homogeneously distributed on the membrane, but rather is organized in nanoclusters^22,23^, while no data are available about the nanoscopic distribution of endogenous α1-NKA. Using super-resolution microscopy, we found that the lack of PRRT2 results in an increased clustering of α3-NKA on the neuronal membrane, due to the aggregation of the smallest nanoclusters to form larger clusters, an effect that is fully rescued by the expression of PRRT2. These results are in line with recent studies showing that clustered and free α3-NKA molecules on the membrane surface are in a dynamic equilibrium via diffusion-dependent mechanisms^15^. Indeed, the interaction of Prrt2 may modulate the α3-NKA membrane distribution and its mobility.

This finding is particularly interesting, since the cluster size may influence α3-NKA function. Indeed, Shrivastava et al. ^23^ have shown that treatment of hippocampal neurons with synuclein fibrils caused a redistribution of α3-NKA on the plasma membrane, with the formation of larger clusters associated with functional impairments of the pump activity^23^. More recently, extracellular molecules interacting with β-NKA subunits were found to increase the size of α3-NKA clusters causing a reduced α3-NKA mobility on the plasma membrane and a lower efficiency in Na^+^ extrusion by the pump^22^. These data indicate that the distribution and size of membrane clusters is crucial for the correct function of α3-NKA. In agreement with this concept, we found that PRRT2 deficiency significantly impairs the NKA response to intense excitatory stimuli associated with Na^+^ entry, such as in response to a glutamate puff or to high-frequency stimulation. Also, the functional impairment in the stimulated pump activity could be normalized by rescue of PRRT2 expression, fully supporting an important role of PRRT2 in the regulation of NKA function. Since α3-NKA has lower Na^+^ affinity compared to α1-NKA and therefore plays a major role during high neuronal activity^17^, the lack of PRRT2 may have a larger impact on α3-NKA, leading to neuronal hyperactivity. These effects of PRRT2 deficiency are in line with the putative function of PRRT2 as a stabilizer of neural network activity^7^.

PRRT2 and ATP1A3 gene mutations are linked to various disorders of excitability and to a spectrum of paroxysmal diseases sharing overlapping phenotypes. α3-NKA mutations account for the majority of cases of AHC, a severe syndrome of infancy characterized by several paroxysmal manifestations including epilepsy, alternating hemiplegia, dystonic attacks (either of one limb, a hemibody, or generalized) and ataxia. Moreover, RDP, characterized by generalized dystonia and parkinsonism with onset in late adolescence or early adulthood, is linked to α3-NKA mutations. Over time, more than 50% of these patients develop epilepsy, in addition to their episodic movement disorders. These clinical manifestations involve diverse brain areas, such as hippocampus, cerebral cortex, basal ganglia and cerebellum, all regions where PRRT2 and α3-NKA are co-expressed^11,50^. Recently, both genes were linked to network alterations in the cerebellum that generate similar dystonic phenotypes^1,11,12,51–55^.

The novel biochemical and functional interaction between PRRT2 and α3-NKA described here highlights the importance of their common role in the control of neuronal excitability and how their disruption could lead to similar pathophysiological manifestations. This scenario opens novel roads to the understanding of the molecular basis for these overlapping phenotypes.

In conclusion, when the reported interactions with ion channels, SNARE proteins and NKA are considered, PRRT2 appears as part of a scaffold system that regulates the activity and membrane exposure of the main players of neuronal excitability. Thanks to its multidomain structure, PRRT2 may not be devoted to a single membrane actuator: rather, it may exert an overall inhibitory control of intrinsic excitability by negatively modulating Na_V_ channel exposure and regulating the distribution and function of NKA pumps on the plasma membrane.

## Materials and methods

### PRRT2 KO mice

PRRT2 KO mice were generated by EUCOMM/KOMP using a targeting strategy based on the ‘knockout-first’ allele^11,56^. Mutant animals in a C57BL/6N background were propagated as heterozygous colonies in the Italian Institute of Technology SPF facility. All experiments were carried out in accordance with the guidelines established by the European Communities Council (Directive 2010/63/EU of 22 September 2010) and were approved by the Italian Ministry of Health (authorization nos. 73/2014-PR and 1276/2015-PR).

### Plasmids

The PRRT2-based constructs used in the study are listed below. PRRT2-HA: pKH3-PRRT2-HA vector encoding for full length PRRT2 with HA tag fused at C terminus^6^; BAP-HA: pKH3-BAP-HA vector^9^; PRRT2ΔN-HA: pKH3-PRRT2ΔN-HA encoding for membrane domain of PRRT2^6^; IFTM1: pCMV-HA-mIFITM1 encoding for the mouse sequence of IFTM1 (a kind gift from Jacob Yount, Addgene plasmid #58415; http://n2t.net/addgene: 58415; RRID:Addgene_58415)^57^; PRRT2ΔC-HA: PRRT2 cytosolic domain that, to avoid degradation, was fused to the second membrane domain of IFTM1, a transmembrane protein that shares with PRRT2 an identical membrane topology.

The corresponding region of mouse IFTM1 cDNA was amplified by PCR (High fidelity Taq Polimerase) from pCMV-HA-mIFITM1 with the primers: forward, ACGTAAGCTTACCGCCAAGTGCCTGAACAT; reverse, TCAGGTCGACTCTAATGGCACAGACAACGATGAC and cloned in Hind3-Sal1 sites of the pKH3 plasmid. The sequence corresponding to the intracellular domain of PRRT2 (nucleotides: 1–798) was PCR amplified from pKH3-PRRT2-HA vector with the primers: forward, CTGAAAGCTTATGGCAGCCAGCAGCTCTC and reverse, GATCAAGCTTTTCGCCCCCTTCCACCCCAGGC and cloned in Hind3 single site of the vector encoding the second transmembrane domain of the IFITM1. The correct orientation and sequence of the cloned fragment were controlled by restriction analysis followed by DNA sequencing.

The α3 and α1 NKA-superecliptic pHluorin (SEP) constructs were generated by Dr. Anita Aperia^22^: SEP is inserted in the NKA extracellular loop between transmembrane domains 3 and 4, flanked with flexible linkers to minimize structural interference. PRRT2 silencing (SH1 and SH4) and control (scramble) shRNA sequences inserted into a pLKO.1-CMV-bicistronic lentiviral vector carrying a Tourquoise reporter have been previously generated and characterized^8,36^. For rescue experiments an Sh-resistant version of mouse PRRT2 fused to the mCherry reporter and inserted in the p743.pCCLsin.PTT.hPGK.GFP.Wpre_mut_AMP lentiviral vector and a Cherry variant control have been previously characterized^8,9^.

### Biochemical assays

#### Mouse brain extracts

Mouse brains were homogenized in ice-cold buffered sucrose solution plus 100 mM NaCl and protease inhibitors and cleared by low speed centrifugation (1000 × *g* for 10 min at 4 °C). The supernatant was centrifuged (12,000 × *g* for 15 min at 4 °C), resuspended and incubated in lysis buffer (1% Triton X-100, 150 mM NaCl, 50 mM Tris–HCl pH 7.4, 1 mM EDTA pH 8 with protease inhibitors cocktail) at 4 °C for 1 h and then centrifuged at 12,000 × *g* for 10 min.

#### Pulldown assays for interactome

COS-7 cells were transfected with either PRRT2-HA or BAP-HA used as a control; after 24 h cells were harvested in lysis buffer. Monoclonal anti-HA-agarose affinity resin (Sigma Aldrich) was incubated with cell extracts according to manufacturer’s instructions at 4 °C for 4 h. After four washes in lysis buffer, immunocomplexes were incubated at 4 °C overnight with total mouse brain extracts to allow binding of potential interacting proteins. Pulled down proteins were eluted by HA peptide solution (200 µg/mL HA-peptide in lysis buffer), subjected to SDS–PAGE on 10% polyacrylamide gels, and processed for Coomassie staining, Western blotting, and mass spectrometry.

#### Co-immunoprecipitation assays

For immunoprecipitation, 5 μg of mouse anti-α3-NKA or anti-α1-NKA or mouse control IgGs (anti-GFP, Millipore) were precoated with Protein G Sepharose (GE Healthcare) overnight and incubated with a mouse brain extract in lysis buffer. After extensive washes in lysis buffer and detergent-free lysis buffer, samples were resolved by SDS–PAGE and subjected to western blotting with anti-PRRT2 and anti-α-NKAs antibodies.

#### Pulldown assays with PRRT2 domains

COS-7 cells were co-transfected with: PRRT2-HA, PRRT2ΔN-HA, PRRT2ΔC-HA, using IFITM1 or BAP-HA as controls, together with either α3-NKA-SEP or α1-NKA-SEP. After 24 h, cells were harvested in lysis buffer and processed as described above.

#### Surface biotinylation

Mouse WT and PRRT2 KO hippocampal neurons at DIV14 were incubated with 1 mg/mL of EZ-Link™ Sulfo-NHS-SS-Biotin in cold phosphate-buffered saline (PBS) at 4 °C for 40 min, with constant mixing. Free biotin was quenched, twice, with 100 mM Tris in cold PBS, and once with cold PBS to remove the excess of biotin. Cells were then harvested with lysis buffer for 30 min. Whole cell lysates were centrifuged at 16,000×*g* at 4 °C for 15 min. One mg of the supernatant was incubated with 100 µL of NeutrAvidin-conjugated agarose beads at 4 °C for 3 h, and the remaining supernatant was kept as the input. The beads were subsequently washed five times with lysis buffer before elution. All reagents were purchased from ThermoFisher Scientific.

#### Western blotting

For western blotting analysis, the sample protein concentration was determined using the BCA or Bradford assay and equivalent amounts of protein were subjected to SDS–PAGE on polyacrylamide gels and blotted onto nitrocellulose membranes (Whatman). Blotted membranes were blocked for 1 h in 5% milk in Tris-buffered saline (10 mM Tris, 150 mM NaCl, pH 8.0) plus 0.1% Triton X-100 and incubated overnight at 4 °C with the appropriate primary antibody^9^. Membranes were washed and incubated at room temperature for 1 h with peroxidase-conjugated secondary antibodies. Bands were revealed with the ECL chemiluminescence detection system (ThermoFisher Scientific)^58^.

### Liquid chromatography–tandem MS (LC–MS/MS) analysis

MS was performed at IFOM Functional Proteomic Center (Milan, Italy). Bands of interest were cut from Coomassie-stained gels, reduced, alkylated, and finally digested overnight with trypsin (Roche) as previously described^59^. After acidification, peptide mixtures were concentrated and desalted on homemade Stagetips µC18^60^, dried in a Speed-Vac and resuspended in 10 µL of 0.1% formic acid. LC–ESI–MS/MS of 5 µL of each sample was performed on a Fourier transformed-LTQ mass spectrometer (FT-LTQ, Thermo Electron, San Jose, CA).

#### Database searching

Tandem mass spectra were extracted by RAW2MSM ver.1.10_2007_06.14, converted into peaklist (.msm) and analyzed using Mascot (Matrix Science, London, UK; version 2.3.02) and X! Tandem (The GPM, thegpm.org; version CYCLONE (2010.12.01.1)) searching against UniProt_CP_Mouse_20131113 database (51,192 entries).

#### Criteria for protein identification

Scaffold (version Scaffold_4.4.3, Proteome Software Inc., Portland, OR) was used to validate MS/MS-based peptides and protein identification. Peptide identifications were accepted if they could be established at >95% probability. Peptides Probabilities from X! Tandem were assigned by the Scaffold Local FDR algorithm. Peptide Probabilities from Mascot (Ion Score Only) were assigned by the Peptide Prophet algorithm^61^ with Scaffold delta-mass correction. Protein identifications were accepted if they could be established at >99% probability and contained at least three identified peptides.

### Cell culture procedures, transient transfections, and transductions

COS-7 cells (ATCC) were cultured in advanced DMEM supplemented with 5% fetal bovine serum, 1% l-glutamine, 100 units/mL penicillin, and 100 g/mL streptomycin (Life Technologies) and maintained at 37 °C in a 5% CO_2_ humidified atmosphere. Cells were transfected with Lipofectamine 2000 (Life Technologies) according to manufacturer’s instructions at 80% confluency. Low-density hippocampal neurons were prepared from WT and PRRT2 KO mice as previously described^62^. Animals were sacrificed by CO_2_ inhalation, and 17/18-day embryos (E17–18) were removed immediately by cesarean section. In brief, hippocampi were dissociated by enzymatic digestion in 0.125% Trypsin for 20 min at 37 °C and then triturated with a fire-polished Pasteur pipette. No antimitotic drugs were added to prevent glia proliferation. Neurons were transfected at 10 DIV using 2 μl of Lipofectamine 2000 and 1 μg of plasmids; after 1 h the medium was removed and replaced with equal volumes of fresh and conditioned medium (1:1). For lentiviral infection experiments, low-density hippocampal neurons were transduced with 4 MOI (multiplicity of infection) of lentiviral vectors added to the cell medium at 6 DIV. After 24 h, the medium was removed and replaced with equal volumes of fresh and conditioned medium (1:1). For rescue experiments in PRRT2 KO neurons, cells were alternatively transduced with the PRRT2-Cherry lentiviruses or with the Cherry-alone lentivirus as a control, while WT neurons were treated with the Cherry control lentivirus only (4 MOI). For rescue experiments in PRRT2 silenced neurons, neurons were co-transduced with lentiviruses expressing either shRNA or Scrambled and lentiviruses expressing either Sh-resistant PRRT2-Cherry or Cherry alone (4 MOI + 4 MOI). All reagents were purchased from ThermoFisher Scientific.

### Immunocytochemistry

#### Live immunolabeling

Hippocampal neurons co-transfected with PRRT2-HA and α1-NKA-SEP (B) or α3-NKA-SEP constructs were live-labeled by diluting primary antibodies (mouse anti-HA, 1:500, Invitrogen; rabbit anti-GFP, 1:500, Millipore) in culture medium for 1 h at 37 °C in a 5% CO_2_ incubator to detect surface epitopes, followed by fixation with 4% paraformaldehyde (PFA) and incubation with Alexa Fluor 488 or 594 secondary antibodies.

#### α3-NKA/α1-NKA immunolabeling

Neurons were fixed in 10% trichloroacetic acid (TCA; Merck) at 4 °C for 10 min, washed in PBS and blocked with 10% BSA for 20 min; samples were sequentially incubated with primary antibodies diluted in 5% BSA (α3-NKA MA3-915 monoclonal antibody from Thermo Scientific, 1:300; α1-NKA a6F monoclonal antibody (1:10, 2.7 g/mL) developed by Douglas M. Fambrough and obtained from the Developmental Studies Hybridoma Bank, University of Iowa), followed by Alexa 564-conjugated or 488-conjugated secondary antibodies (Invitrogen; 1:200) at room temperature. After several washes in PBS, coverslips were mounted using Prolong Gold antifade reagent (Invitrogen) containing DAPI (4′,6′-diamidino-2-phenylindole) for nuclear staining.

#### Dual step immunolabeling

An alternative immunofluorescence protocol, optimizing and combining the protocols described for single proteins^10,17^, was applied to stain endogenous α3-NKA/α1-NKA and PRRT2. Primary hippocampal neurons were fixed in 4% PFA, 4% sucrose in PBS for 20 min, permeabilized with 0.1% Triton X-100 in PBS for 5 min and incubated with 5% bovine serum albumin (BSA) and 5% fetal bovine serum in PBS (blocking solution) for 1 h. Samples were incubated with the primary antibody (anti-PRRT2, a kind gift of Dr. J.-W. Tsai, 1:200) in blocking solution at 4 °C overnight. Neurons were post-fixed in 10% TCA (Merck) at 4 °C for 10 min and processed for immunolabeling with α3-NKA/α1-NKA antibodies as described above.

### Microscopy and image analysis

Confocal imaging was performed on a Leica TCS SP5 AOBS TANDEM confocal microscope. Confocal scanning was done with a ×63/1.4 APO L W UVI objective using the Leica LAS AF software system. ImageJ JACoP plugin^63^ was used to quantify colocalization by calculating the percentage of colocalization based on the Manders’ overlapping coefficient^64^.

Structural illumination microscopy (SIM) imaging was performed on Nikon N-SIM with technology 3DSIM^65^ using CFI Apo TIRF 100×C Oil N.A. 1.49 objective. Software Nikon NIS-Elements with SIM module was set up to imaging reconstruction, with following parameters: IMC—illumination modulation contrast 5; HRNS—high-resolution noise suppression: 0.10; 0.15 (488;561); OFBS—out of focus blur suppression: 0.15. We performed all the STED super-resolution images on a Leica TCS SP5 gated-STED microscope, using an HCX PL APO 100 ×100/1.40/0.70 oil immersion objective lens (Leica Microsystems, Mannheim, Germany). Emission depletion was accomplished with a 592 nm STED laser, a white laser provided the desired wavelength of 488 nm for Alexa fluortm_488 excitation. The fluorescence emission was detected at 500–580 nm, with 1.30–10 ns time gating and gain 500% using a hybrid detector (Leica Microsystem). We acquired images at 8000 Hz by resonant scanning with 128 line average and pixel size of 25 nm. We estimate our resolution better than 55 nm using the intensity profile analysis of sub-resolved-sized structures by measuring the full-width-half-maximum (FWHM) of 2D Gaussian curve fitting^66–68^.

The analysis of α3-NKA clusters in primary hippocampal neurons was performed on over 35 images (3D-SIM) and 25 (STED) of WT and PRRT2 KO neurons (DIV 14-17) from three independent preparations. Fiji ImageJ software was used to process images and analyze the area and number of α3-NKA clusters. For each image, regions of interest (ROI) were drawn along the soma or neurite membrane. For WT, PRRT2 KO and PRRT2 KO + PRRT2 neurons, threshold plugin was applied, setting up the same score to isolate the fluorescent particles. The plugin generates a binary image that was analyzed via the “analyze particles” function. A minimum size cut-off of 0.002 μm^2^ was applied to eliminate speckled background stain. For each image, clusters were divided in bins based on their size (3D-SIM: <140; 141–190; 190–300 nm; STED: <70; 71–100; 101–120; 121–140; 141–160; 161–220 nm) the percent of cluster area and number occupied by each group on the total clusters area and number, respectively, was calculated for each genotype.

### Live imaging for NKA membrane expression and trafficking

α3-NKA-SEP or α1-NKA-SEP were transfected in WT and PRRT2 KO neurons at 10 DIV as previously described. At 12–14 DIV, neurons were mounted into imaging chambers (Quick Exchange Platform; Warner Instruments), and sequentially perfused with a laminar flow system with Tyrode solution (140 mM NaCl, 3 mM KCl, 2 mM CaCl_2_, 1 mM MgCl_2_, 10 mM HEPES, 10 mM glucose, pH, 7.4) followed by Tyrode solution at pH 5.5 buffered with 10 mM MES and by perfusion with 50 mM NH_4_Cl–Tyrode solution. Epifluorescence records were collected using an Olympus IX-81 microscope equipped with an MT20 Arc/Xe lamp with a ×60 magnification objective, on the Excellence’RT software (Olympus). Linear ROIs at the plasma membrane were drawn and fluorescent intensity along time analyzed by ImageJ (3-pixel width)^8^. Membrane expression was calculated at plasma membrane ROIs, where the fluorescence in Tyrode (*F*_0_) represents total fluorescence (specific membrane expression signal plus background), while the fluorescence in the extracellular acidic MES buffer (F_MES_) represents the background signal only. Therefore, (*F*_0_ − F_MES_)/F_MES_ allows to estimate the specific membrane expression at the membrane ROIs.

### Electrophysiological recordings

Whole-cell patch-clamp recordings were conducted in low-density hippocampal neurons at 10–14 DIV. All experiments were performed at 22–24 °C. Recordings were conducted using borosilicate glass (Kimble, Kimax, Mexico) microelectrodes pulled to a final resistance of 3-4 MΩ when filled with a standard internal solution that contained (in mM): 126 K gluconate; 4 NaCl, 1 MgSO_4_, 0.02 CaCl_2_, 0.1 BAPTA, 15 glucose, 5 HEPES, 3 ATP, and 0.1 GTP (pH 7.3 with KOH). Cultures were continuously perfused with an external recording solution containing (in mM): 140 NaCl, 2 CaCl_2_, 1 MgCl_2_, 4 KCl, 10 glucose, 10 HEPES (pH 7.4 with NaOH). An EPC-10 amplifier (HEKA Electronic) was used in either current-clamp or voltage-clamp configuration. Data acquisition was performed using the PatchMaster program (HEKA Electronic). Series resistance was compensated 80% (2 µs response time) and the compensation was readjusted before each stimulation. Recordings with leak currents >200 pA or series resistance >20 MΩ were discarded. Electrophysiological data were analyzed using FitMaster (HEKA Electronic) and Origin (Microcal Software, Northampton, MA, USA) softwares.

### Analysis of the NKA activity evoked by glutamate

Voltage-clamp recordings were acquired at 20 kHz and low-pass filtered at 4 kHz. The external solution contained 10 µM CGP55845 and 30 µM bicuculline to block GABA_B_ and GABA_A_ receptors, respectively, 50 µM D-AP5 to limit NKA inhibition by Ca^2+^ entry through activated NMDA receptors^29,30^ and 1 µM TTX to block spontaneous APs. CNQX was excluded from the solution to allow Na^+^ entry through AMPA receptor activation. All drugs were purchased from Tocris Bioscience (Bristol, UK). A transient increase in the intracellular Na^+^ concentration was induced focally by applying glutamate (250 µM) through a gravity system constituted by a multi-barreled pipette with a single outlet and three inlets controlled by electrovalves (Warner Istruments) operated by a PC. The perfusion solution could be changed rapidly (30 ± 6 ms) and short puffs of the stimulating solution were applied for controlled periods of time (250 ms). The tip of the perfusion pipette (40 ± 5 µm) was placed close to the soma (20 ± 4 µm) of the recorded neuron.

The glutamate puff elicited a 0.5–1.5 nA inward current lasting 5–10 s. To ensure consistency of the evoked responses, the stimulation was delivered every 30 s and the results of three consecutive trials were averaged. Glutamate-induced NKA activity was measured by digitally subtracting the glutamate response obtained in the presence of 1 mM ouabain (Sigma-Aldrich) from the response obtained under control conditions. The integration of the resulting ouabain-sensitive current yielded the NKA charge^30^.

### Analysis of the post-train AHP potential

Current-clamp recordings were sampled at 50 kHz and filtered at 1/5 of the acquisition rate with a low-pass Bessel filter. Cells were maintained at a holding potential of −70 mV, in an external solution supplemented with 50 µM D-AP5, 10 µM CNQX, 10 µM CGP55845, and 30 µM bicuculline, to block NMDA, non-NMDA, GABA_A_, and GABA_B_ receptors, respectively. Post-tetanic AHP was evoked by stimulating neurons with a train of 80 APs at 50 Hz^34^. The current value used to evoke APs was selected as the minimal current able to evoke an AP for each step of the train when applied at 10 Hz, increased by 100 pA^69^. The AHP was recorded for 20 s after the end of the train stimulation. To examine the AHP component generated by NKA, the protocol was repeated in the presence of 1 mM ouabain. The ouabain-induced depolarization was compensated by injecting an additional depolarizing current in order to maintain a holding potential of −70 mV. AHP integrals were determined as the areas under the AHP waveforms, baselined to −70 mV and within a time window of 20 s after the end of the last current step. The magnitude of the NKA-dependent AHP (NKA-AHP) was obtained by subtracting the control AHP area from the area of the AHP generated in the presence of ouabain^33,34^.

### Statistical analysis

Data are expressed as means ± standard error of the mean (SEM) for number of cells (*n*) or mouse preparations, as detailed in the figure legends. Normal distribution of data was assessed using the D’Agostino–Pearson’s normality test. The *F*-test was used to compare variance between two-sample groups. To compare two normally distributed sample groups, the unpaired or paired two-tailed Student’s *t*-test was used. To compare two-sample groups that were not normally distributed, the Mann–Whitney’s *U*-test was used. To compare more than two normally distributed sample groups, one-way ANOVA, followed by post hoc multiple comparison tests was used. Alpha levels for all tests were 0.05% (95% confidence intervals). Statistical analysis was carried out using OriginPro-8 (OriginLab Corp., Northampton, MA, USA) and Prism (GraphPad Software, Inc.) software.

## Supplementary information

Suppl. Fig 1

Suppl. Figure 2

Suppl. Figure 3

Suppl. Figure 4

Suppl. Figure 5

Suppl. Table 1

Suppl Figure Legends
