# Supplementary figures and images for: An interaction between PRRT2 and Na+/K+ ATPase contributes to the control of neuronal excitability
Source: Cell Death Dis. 2021 Mar 17;12(4):292. doi: 10.1038/s41419-021-03569-z (PMC7969623; doi:10.1038/s41419-021-03569-z)

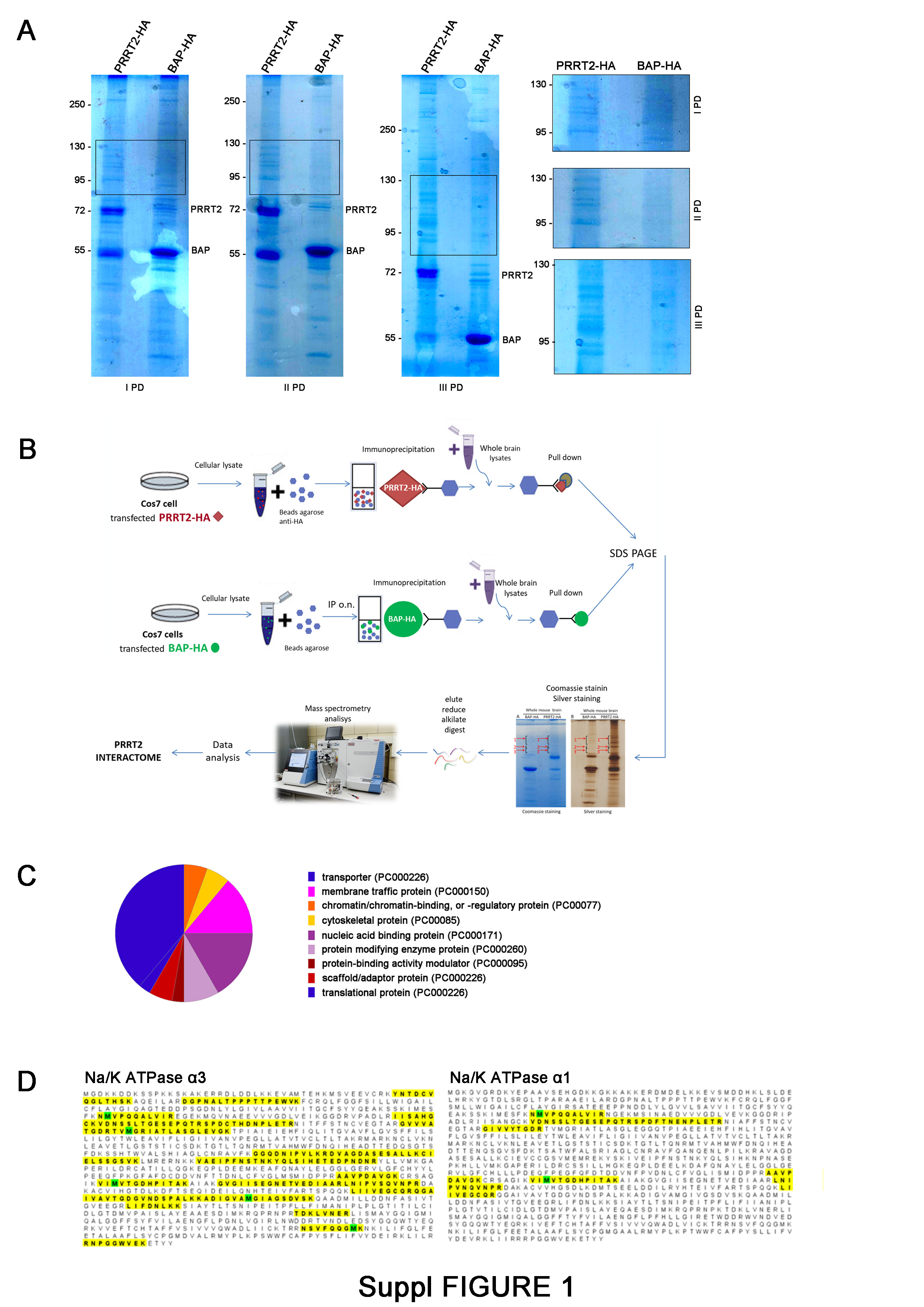

Supplement: Supplementary file 1 — Suppl. Fig 1 [file 41419_2021_3569_MOESM1_ESM.jpg]

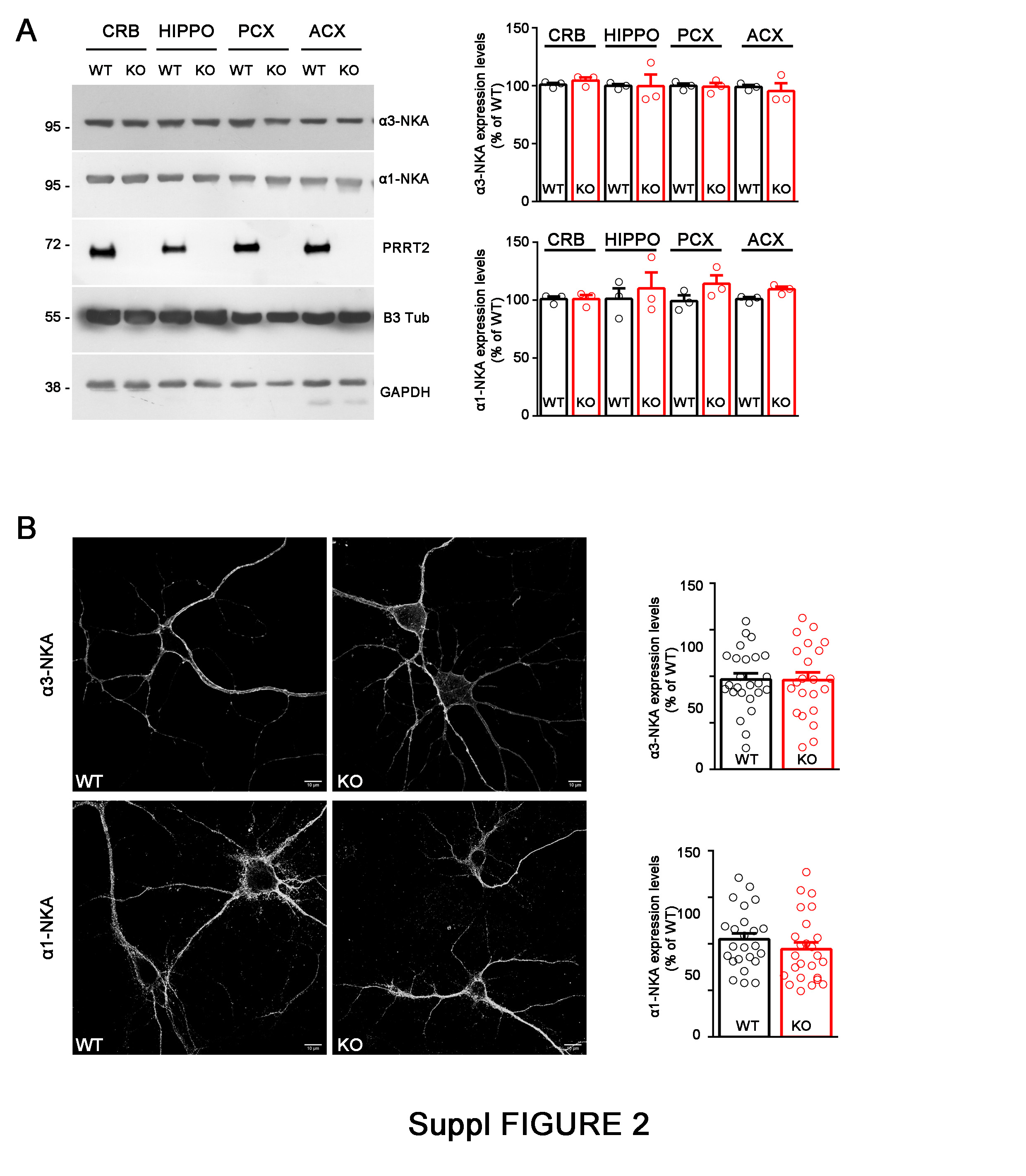

Supplement: Supplementary file 2 — Suppl. Figure 2 [file 41419_2021_3569_MOESM2_ESM.jpg]

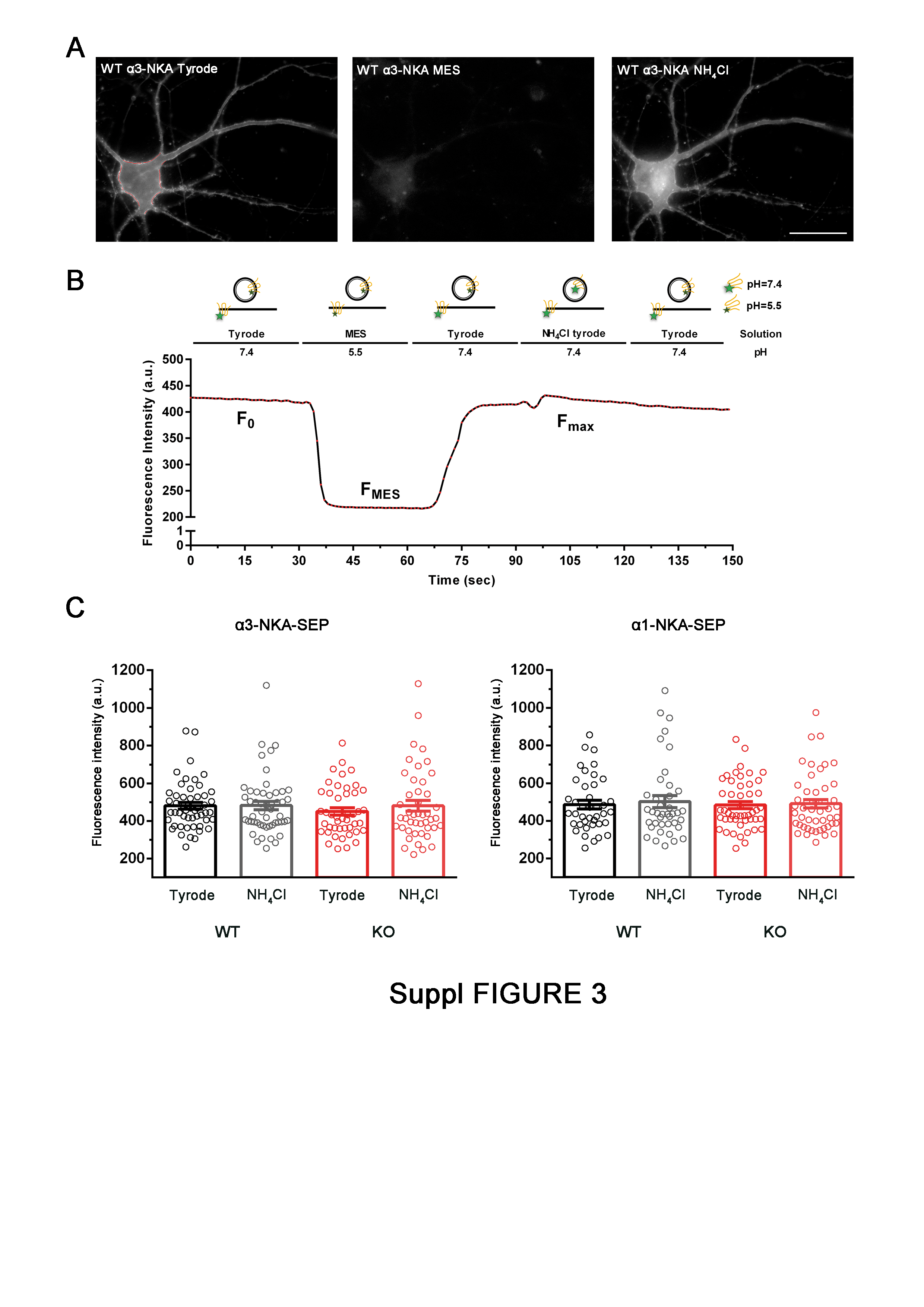

Supplement: Supplementary file 3 — Suppl. Figure 3 [file 41419_2021_3569_MOESM3_ESM.png]

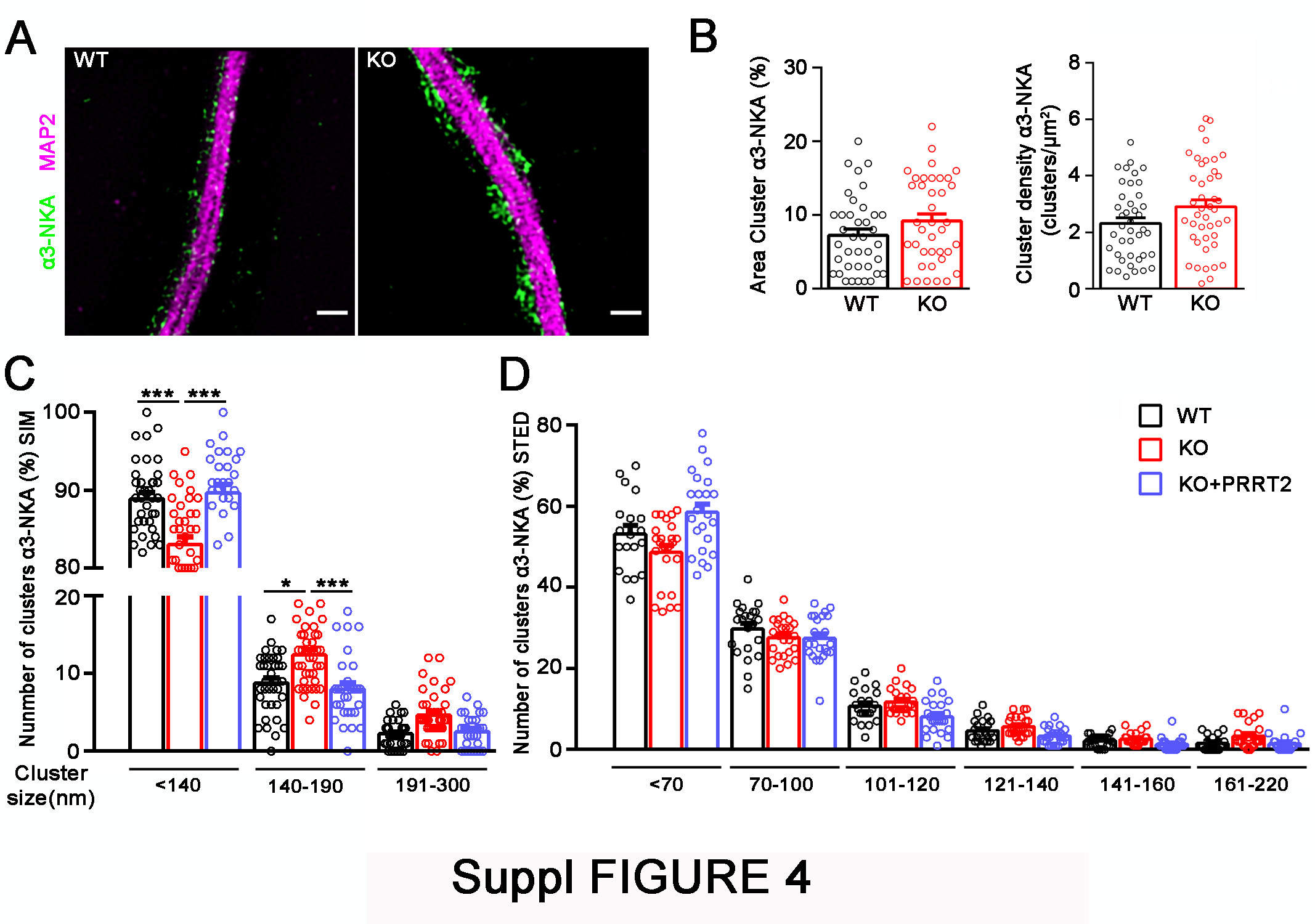

Supplement: Supplementary file 4 — Suppl. Figure 4 [file 41419_2021_3569_MOESM4_ESM.jpg]

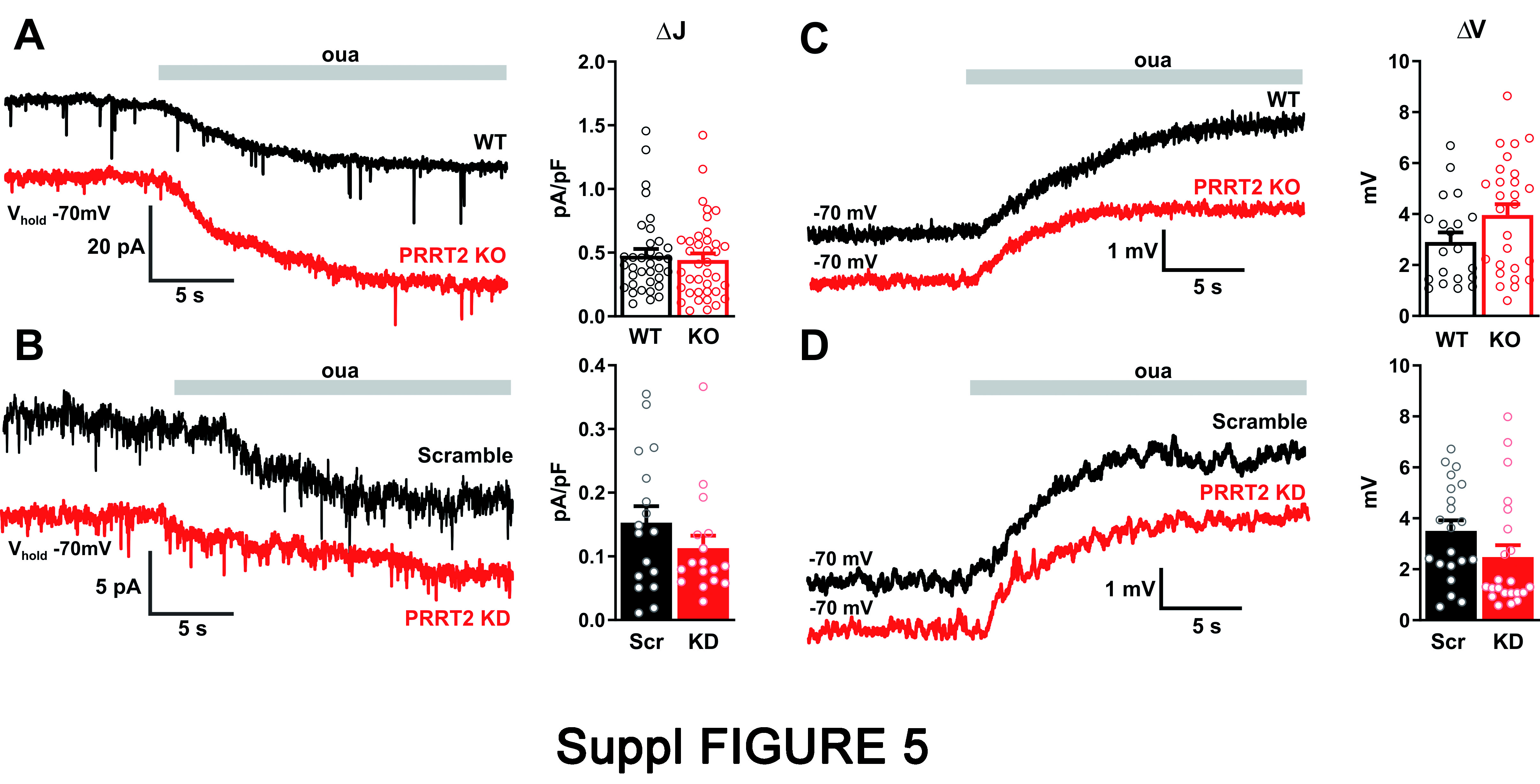

Supplement: Supplementary file 5 — Suppl. Figure 5 [file 41419_2021_3569_MOESM5_ESM.jpg]
