# Supplementary material for: An interaction between PRRT2 and Na+/K+ ATPase contributes to the control of neuronal excitability
Source: Cell Death Dis. 2021 Mar 17;12(4):292. doi: 10.1038/s41419-021-03569-z (PMC7969623; doi:10.1038/s41419-021-03569-z)
Supplement: Supplementary file 7 — Suppl Figure Legends [file 41419_2021_3569_MOESM7_ESM.docx]

**LEGENDS TO THE SUPPLEMENTARY FIGURES**

**Supplementary Figure 1. Identification of new PRRT2 interacting proteins by proteomics and functional classification of the most significant hits. (A)** Coomassie blue stained SDS-PAGE gels of proteins affinity-purified by pulldown (PD) with either PRRT2-HA or BAP-HA from extracts of total mouse brain. Selected bands (95-130 kDa inset) were excised from the gels and analyzed by Liquid Chromatography Mass Spectrometry. This region included several differences in the banding pattern between PRRT2-HA and the BAP-HA control that were present in 3 separate experiments (I-III PD). (**B**)Proteomic workflow for downstream Liquid Chromatography Mass Spectrometry. PRRT2-HA and BAP-HA were expressed in Cos7 cells. After cells lysis, baits were immunoprecipitated by monoclonal anti-HA-agarose affinity resin and subsequently incubated with mouse whole brain extracts. The immunocomplexes composed of baits and interactors were run on polyacrylamide gels and processed for Coomassie staining. Selected areas of the gel were processed and analyzed by Liquid Chromatography Mass Spectrometry. **(C)** Functional classification of the interactors identified by Mass Spectrometry according to the protein class. The pie chart was generated by PANTHER Classification system. Protein class name (Gene Ontology class ID). (**D**) α3-NKA and α1-NKA amino acid sequences. The exclusive spectra sequences identified by mass spectrometry analysis are highlighted in yellow.

**Supplementary Figure 2. Total expression of** **α3-NKA and α1-NKA proteins is not altered in PRRT2 KO neurons. (A)** Unaltered expression of α3-NKA and α1-NKA in various regions of WT and PRRT2 KO mouse brains. *Left:* Representative immunoblots. GAPDH was used as a control for equal loading. *Right:* Densitometric analysis of the immunoblots. The expression level of α3-NKA and α1-NKA in PRRT2 KO brain areas, calculated in percent of the respective value in the WT, is shown as means ± SEM of n=3 independent experiments; unpaired Mann-Whitney’s *U*-test. Abbreviations: CRB, cerebellum; HIPPO, hippocampus; PCX, posterior cortex; ACX, anterior cortex. **(B)** *Left:* Representative confocal images of WT and PRRT2 KO hippocampal neurons (DIV14-17) stained for α3-NKA (*top*) and α1-NKA (*bottom*). Scale bar, 10 µm. *Right:* The histograms (means ± SEM) show the absence of significant differences in the mean fluorescent intensity between WT and PRRT2 KO cells. Data refer to n =30 neurons per genotype, from n=3 independent preparations (2 coverslips/preparation/genotype); unpaired Student’s *t*-test.

**Supplementary Figure 3. Experimental protocol in NKA-SEP transfected neurons. (A)** Representative images of a WT neuron transfected with α3-NKA-SEP during Tyrode, MES and NH_4_Cl-Tyrode perfusion showing the ROIs drawn for the evaluation of NKA expression at the plasma membrane (red). Scale bar, 20 μm. **(B)** Schematic representation of the experimental live imaging protocol. A cartoon depicting the SEP state under the various environmental conditions is shown on the top. Black lines and circles are representative of the plasma membrane and intracellular compartments, respectively. The graph shows the time course of fluorescence changes in a representative membrane ROI, as shown in A, with measurable values for F_0_, F_MES_ and F_max_. **(C)** The absence of detectable changes in α3-NKA-SEP (*left*) or α1-NKA-SEP (*right*) fluorescence during perfusion with Tyrode and NH_4_Cl-Tyrode proves the reliability of the ROI selection for the membrane pool of NKA.

**Supplementary Figure 4. (A)** Representative 3D-SIM images of hippocampal neurons (DIV 14-17) stained for α3-NKA (green) and counterstained with MAP2 antibodies (magenta) for the identification of soma and dendrites. **(B)** *Left*: Total area (mean ± SEM) of α3-NKA nanoclusters expressed in percent of the ROI area. *Right*: Density (mean ± SEM) of α3-NKA nanoclusters per µm^2^ obtained by counting the total number of clusters. Data refer to n = 50 neurons per genotype, from n = 3 independent preparations (2 coverslips/ preparation/genotype). Scale bar, 1 μm. (**C,D**) Distribution of the number of α3-NKA membrane clusters by 3D-SIM (**C**) and STED (**D**). Clusters were divided into bins based on their size, as indicated. The histograms (means ± SEM) show the number of clusters in each bin expressed in percent of the total number of α3-NKA clusters. The analysis by 3D-SIM (**C**) shows a significant increase in the number of larger clusters and a reciprocal decrease in the number of smaller clusters along the soma and the dendritic membrane in PRRT2 KO neurons (red bars) with respect to WT neurons (black bars). The change was fully rescued by expression of PRRT2 in PRRT2 KO neurons (KO+PRRT2, blue bars). Data refer to n = 35 (3D-SIM) and 25 (STED) neurons per genotype, from n=3 independent preparations (2 coverslips/preparation/genotype). *p<0.05, ***p<0.001, one-way ANOVA/Bonferroni’s tests.

**Supplementary Figure 5. Resting NKA activity is unaffected in PRRT2 KO and PRRT2 KD neurons. (A)** *Left:* Representative current traces showing the tonic current in response to the application of 1 mM ouabain in WT and PRRT2 KO neurons. *Right:* Bar plot showing means ± SEM of the tonic current inhibited by ouabain, normalized to cell capacitance [current density, J (pA/pF)] for the two experimental groups (n =35 and 38 for WT and PRRT2 KO neurons, respectively, from 3 independent neuronal preparations). **(B)** *Left:* Representative voltage-clamp traces obtained from the protocol as in (A) applied to representative Scr and PRRT2 KD neurons. *Right:* Bar plots of the mean ± SEM inward current density elicited by ouabain treatment with superimposed individual experimental points (n = 17 for both Scr and PRRT2 KD, from 3 independent neuronal preparations). **(C)** *Left:* Representative voltage recordings of the membrane depolarization elicited by the pharmacological blockade of NKA from a holding potential of -70 mV in WT and PRRT2 KO neurons. *Right:* Bar plots of the mean ± SEM membrane depolarization measured in response to ouabain for the two groups (n = 21 and 27 for WT and PRRT2 KO neurons, respectively, from 3 independent neuronal preparations). (**D**) *Left:* Representative current-clamp traces from Scr and PRRT2 KD neurons obtained from the protocol as in (C). *Right:* Bar plots of the mean ± SEM amplitude of the ouabain-induced membrane depolarization in Scr and PRRT2 KD neurons with superimposed individual experimental points (n = 21 and 24 for Scr and PRRT2 KD neurons, from 3 independent neuronal preparations).

**Suppl. Table 1. List of PRRT2 putative interacting proteins obtained by mass spectrometry analysis.** The numbers in the “Hits” column refer to the mean amounts of unique peptides identified in three independent experiments.
